# Molecular Evaluation of Different Enrichment Methods for Extracellular Vesicles from Healthy Subjects’ Biobanked Serum

**DOI:** 10.3390/ijms27020892

**Published:** 2026-01-15

**Authors:** Michela Deiana, Elisabetta Vezzelli, Cristina Mazzi, Denise Lavezzari, Marcello Manfredi, Francesca Moretta, Chiara Piubelli, Federico Giovanni Gobbi, Natalia Tiberti

**Affiliations:** 1 Department of Infectious, Tropical Diseases and Microbiology, IRCCS Sacro Cuore Don Calabria Hospital, 37024 Negrar di Valpolicella, Italy; michela.deiana@sacrocuore.it (M.D.);; 2 Clinical Research Unit, IRCCS Sacro Cuore Don Calabria Hospital, 37024 Negrar di Valpolicella, Italy; 3 Department of Translational Medicine, University of Piemonte Orientale, 28100 Novara, Italy; 4 Institute for Molecular and Translational Cardiology (IMTC), IRCCS Policlinico San Donato, 20097 Milano, Italy; 5 Clinical Analysis Laboratory and Transfusional Medicine, Clinical Pharmacology, IRCCS Sacro Cuore Don Calabria Hospital, 37024 Negrar di Valpolicella, Italy; 6 Department of Clinical and Experimental Sciences, University of Brescia, 25121 Brescia, Italy

**Keywords:** extracellular vesicles, Biobank, serum, proteomics, miRNA, surface phenotyping

## Abstract

Extracellular vesicles (EVs) from human body fluids are valuable tools for biomarker discovery and for exploring the mechanisms underlying various pathologies, including infectious diseases. The translation of EV research into clinical practice is however hindered by the variability in EV pre-clinical investigations. Therefore, standardisation of analytical procedures and reporting policies is essential. Human serum is a key biological matrix for biomarker discovery and is commonly stored within biobanks. Here, we investigated different strategies for EV enrichment from small volumes of biobanked serum and evaluated their impact on EVs’ downstream analyses. EVs were obtained from 250 μL of biobanked serum using ultracentrifugation (UC), size-exclusion chromatography-based methods (ExoSpin-ES, qEV1-35 nm, and qEV1-70 nm), or ExoRNeasy (ER). The resulting EVs were subsequently characterised for morphology, concentration, surface phenotype, and multi-omics profiles. All methods successfully enriched small EVs expressing tetraspanins on their surface, although at different concentrations. The most efficient method for proteomics analyses was qEV1-70 nm, followed by ES, which was more susceptible to contamination by serum proteins. EV-miRNA cargo was effectively profiled in UC-, ES-, and ER-EVs, with the latter providing the broadest miRNA coverage. Our results support the feasibility of using biobanked serum for EV-based research and further highlight the importance of selecting appropriate EV enrichment methods, since they influence both miRNA and protein cargo characterisation.

## 1. Introduction

Extracellular vesicles (EVs) are recognised as an important source of biomarkers, innovative therapeutic vehicles, and key players in disease pathophysiological mechanisms [1,2]. Moreover, within the field of infectious diseases, EVs contribute to host–pathogen interaction through their role in the inter-parasite and inter-kingdom communication, being released by both host cells and pathogens [3,4,5,6].

EVs include a number of particle subtypes that differ in their biogenesis, cargo, and surface markers. Historically referred to as microvesicles (or microparticles or ectosomes) and exosomes, it is now considered more appropriate to use the general term EVs, due to the difficulties in specifically enriching a single population [7,8]. EVs are membrane-enclosed nanoparticles with different size ranges, although overlapping. Small EVs have a diameter between 50 and 150 nm, mostly—but not exclusively—generated within the intracellular multivesicular body (MVB) and released via the endocytic pathway. Small EVs are considered the most abundant in biological fluids. Medium/large EVs are larger particles of 150–800 nm in diameter, originating at the cell plasma membrane [7]. A number of additional non-vesicular nanoparticle structures that might overlap with EVs for some of their biophysical properties are progressively being described, such as exomeres and supremeres [9].

Blood and its derivatives are the most frequently used body fluids for liquid biopsies [10] and represent key sources of EVs. Along with plasma, serum is the most widely used biological matrix in clinical diagnostics and is amongst the most commonly biobanked blood derivatives [11]. Many research institutions are now equipped with regulated biobanks for long-term storage of different samples. In the healthcare context, biobanks have become valuable resources for supporting large multi-centre studies, while ensuring the association of biobanked samples with clinical data and the respect of the privacy and ethical regulations [12]. Moreover, the creation of international networks, such as the Pan-European Biobanking and Biomolecular Resources Research Infrastructure (BBMRI), is becoming instrumental in supporting large-scale studies in many fields, including infectious diseases [13].

According to a recent survey by the International Society for Extracellular Vesicles (ISEV), the number of studies investigating serum-derived EVs has markedly increased in recent years [14], further supporting the growing importance of this biofluid in EV research. However, studying EVs from biobanked serum remains challenging due to numerous technical and biological variables that can affect experimental procedures and consequently the observed results [15,16]. Pre-analytical factors, such as sample processing and storage, can affect carryover of proteins, cell contaminants in EV preparations, or EV integrity [17,18]. Indeed, depending on the sample type (e.g., plasma, serum, platelet-poor plasma), residual cells such as red blood cells, platelets, or leukocytes can release EVs ex vivo, potentially confounding the results [19,20]. In the attempt to standardise and enhance the reproducibility of EV studies using blood derivatives, ISEV recommends improving data reporting and methodological transparency through different tools [16,21,22]. Compliance with this good-reporting practice may increase the reliability and utility of biobanked serum as a source of EVs for pre-clinical investigations.

The study of serum-derived EVs can be of interest for multiple applications, spanning from biomarker discovery, understanding disease pathobiology, or identification of targets for novel diagnostic/therapeutic interventions [23]. Experimental EV-enrichment methods should therefore be carefully selected according to the intended downstream analyses and research objectives, as they can influence EV yield and purity [24,25]. However, to date, no single enrichment technique is able to achieve the highest particle recovery (i.e., particle number) and the highest specificity simultaneously in terms of particle sub-types, as summarised elsewhere [26].

Different comparative studies of EV enrichment methods are available in the literature [24,25,27,28]. However, to the best of our knowledge, they either employed larger sample volumes—thereby limiting their applicability to biobanked specimens—or evaluated only a limited set of morphological or molecular properties of the enriched EVs. We thus deemed it necessary to assess the suitability of small serum volume (i.e., 250 μL) from biobanked collections for pre-clinical EV research by comparing multiple enrichment strategies and examining their impact on EVs surface phenotyping by flow cytometry, as well as on EVs protein and miRNA cargo using multi-omics approaches. In the multi-omics era, integrating multiple discovery approaches is becoming a powerful strategy for biomarker identification. Here, we show that, for EV derived from biobanked serum, the selection of the enrichment method substantially affects the downstream EV molecular characterisation.

## 2. Results

**Figure 1:**
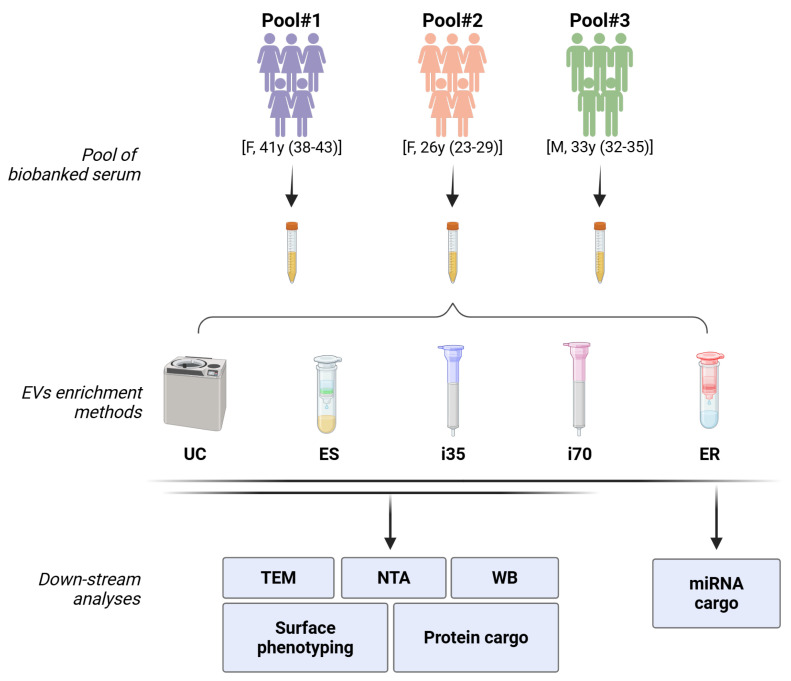
Study design. The sex and median age (with range, in years) of the five healthy subjects whose serum was used to create the three pools are reported within square brackets. UC: ultracentrifugation; ES: Exo-spin^TM^; i35: Izon qEV1-35 nm; i70: Izon qEV1-70 nm; ER: ExoRNeasy; TEM: transmission electron microscopy; NTA: nanoparticle tracking analysis; WB: Western blot. Created with https://BioRender.com.

A summary of the study design is reported in Figure 1. EVs from three pools of biobanked serum from healthy subjects (250 μL each) were investigated using five different enrichment methods, i.e., ultracentrifugation (UC), Exo-spin^TM^ (ES), Izon qEV1-35 nm (i35) and Izon qEV1-75 nm (i70) columns, and ExoRNeasy (ER). The latter was only employed for downstream Next-Generation Sequencing (NGS) analyses since it directly enriches vesicular RNA. The use of pooled samples was necessary to compare the different enrichment methods and the various downstream analyses using the same biological samples. Nonetheless, every procedure was conducted using 250 μL of the sample.

### 2.1. UC, ES, i35, and i70 Enrich Heterogeneous Populations of Small EVs from Biobanked Serum

**Figure 2:**
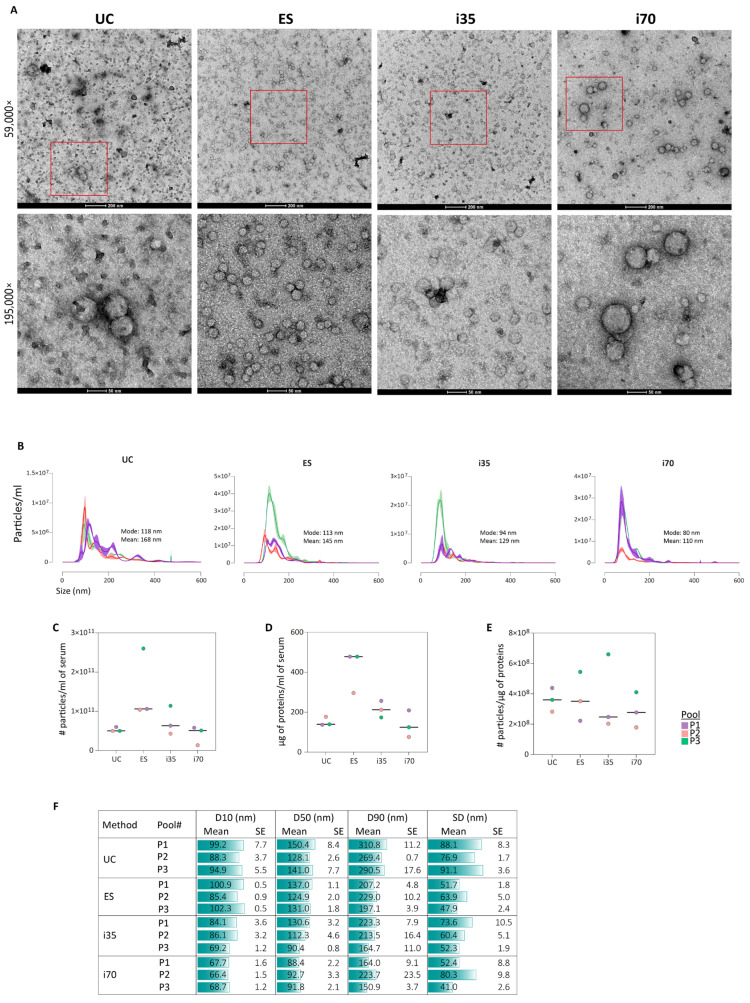
Morphology and size distribution of EVs. (**A**) TEM images of EVs enriched with the different methods. Areas within red squares are visualised at a higher magnification (i.e., 195,000×) in the lower panels. Scale bars: upper panels 200 nm, lower panels 50 nm. (**B**) Profile of the size distribution and concentration of EVs enriched with the different methods in the three biological samples analysed. Purple line: Pool#1; Pink line: Pool#2; Green line: Pool#3. (**C**–**F**) Summary statistics of NTA measurements. (**C**) Number of particles per ml of serum used for enrichment. (**D**) Amount of proteins (μg of proteins per ml of serum used for enrichment) recovered from each preparation as determined by Qubit protein assay. (**E**) Particle-to-protein ratio as an indicator of the purity of the preparation. Black lines in (**C**–**E**) represent the median. (**F**) Size distribution statistics. D10, D50, D90: value of size distribution containing 10%, 50%, or 90% of the measures, respectively. SD: width of the size distribution in nm, i.e., standard deviation of the size distribution profile. For each parameter, the mean and standard error (SE) of the three replicate NTA acquisitions are reported.

All methods enriched particles exhibiting morphology typical of EVs with a size range between 80 and 150 nm (Figure 2).

Imaging by TEM revealed heterogeneous populations among the different samples (Figure 2A), particularly for i70-EVs, in agreement with Veerman et al. [27]. UC-EVs showed a more prominent background signal, likely due to the co-isolation of serum proteins. Despite variability in size, TEM images consistently confirmed that all four methods enriched particles with the typical morphology of EVs. Statistical analysis of EV size distribution and concentration was obtained by NTA (Figure 2B). The i35 and i70 methods enriched EV populations of similar size, with mode diameters of 94 nm and 80 nm, respectively, while UC and ES enriched slightly larger EVs, with mode diameters of 118 nm and 113 nm, respectively. The highest particle count was observed in ES preparations, which also provided the highest amount of proteins (Figure 2C,D). Since protein concentration was determined in non-lysed EV samples, the higher protein concentration might account for contaminant serum proteins. Indeed, when the particle-to-protein ratio was evaluated as an indicator of the purity of the preparation [28], comparable results were obtained across the four methods, with i35-EVs displaying slightly lower purity (Figure 2E). Lastly, considering the spread of the size distribution as an indicator of EV size heterogeneity, UC and i70 displayed broader size distribution profiles (Figure 2F).

### 2.2. EV Enriched from Biobanked Serum Are Positive for Vesicular Markers

**Figure 3:**
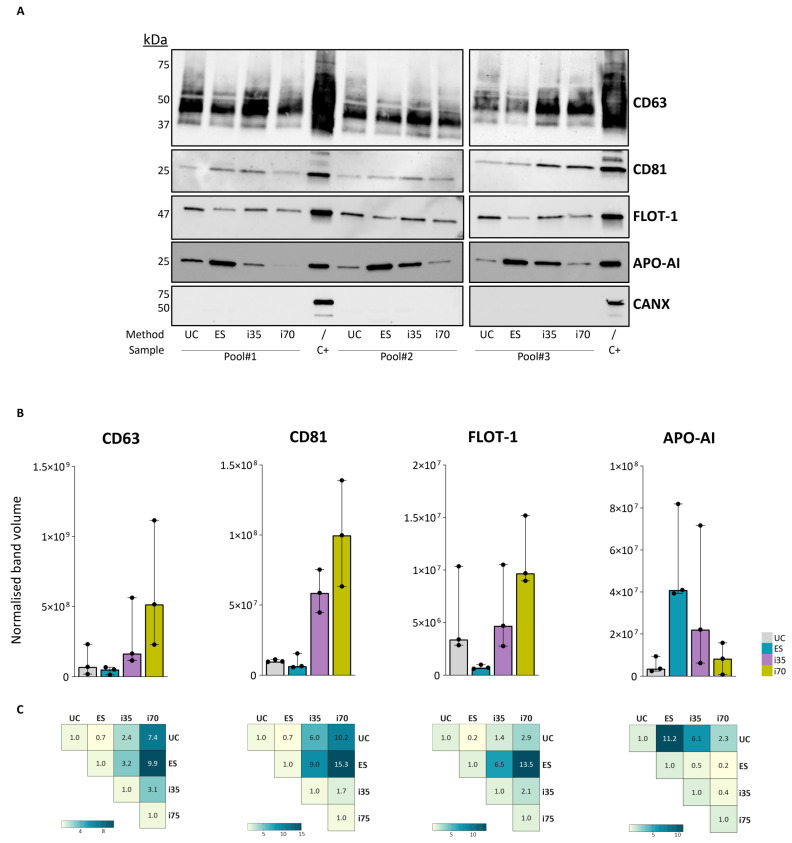
Expression of classical EV markers as determined by Western blot. (**A**) Chemiluminescent images of all assessed targets (CD63, CD81, Flotillin-1, Apolipoprotein AI, and calnexin). Entire WB images are provided as Supplementary Figure S2. (**B**) Band quantification with respect to total load. Bars represent the median normalised band volume with range. (**C**) Fold-change of the median normalised band volume computed to compare the abundance of the different markers according to the EV isolation method. Fold-changes were computed considering the methods reported at the top of the graph as numerators. FLOT-1: flotillin-1; Apo-AI: apolipoprotein AI; CANX: calnexin. Positive controls (C+) were human Caco-2 cell lysate for CD63, CD81, FLOT-1, and CANX; human serum for Apo-AI.

Western blot (WB) was performed to assess the protein composition of the different EV preparations by detecting specific protein targets. All samples were tested for two surface proteins, CD63 and CD81, and one intravesicular protein, flotillin-1 (FLOT-1). In addition, apolipoprotein A-I (APO-AI) was evaluated to assess the degree of contamination from serum proteins, and calnexin (CANX), an endoplasmic reticulum protein typically absent from EVs, was used as a negative control (Figure 3 and Supplementary Figure S2).

CD63 and CD81—multi-pass transmembrane proteins typical of small EVs—were more abundant in i35- and i70-EVs. FLOT-1, a cytosolic protein involved in vesicular trafficking [29], was detected at very low levels in ES-EVs, while it was 5- to 13.5-fold more abundant in EVs enriched with the other three methods. APO-AI exhibited the highest relative abundance in ES preparations, followed by i35, whereas only minimal APO-AI was detected in UC- and i70-EVs. As expected, all EV samples were negative for CANX.

### 2.3. EV Enrichment Methods Partly Affect EV Surface Phenotype

**Figure 4:**
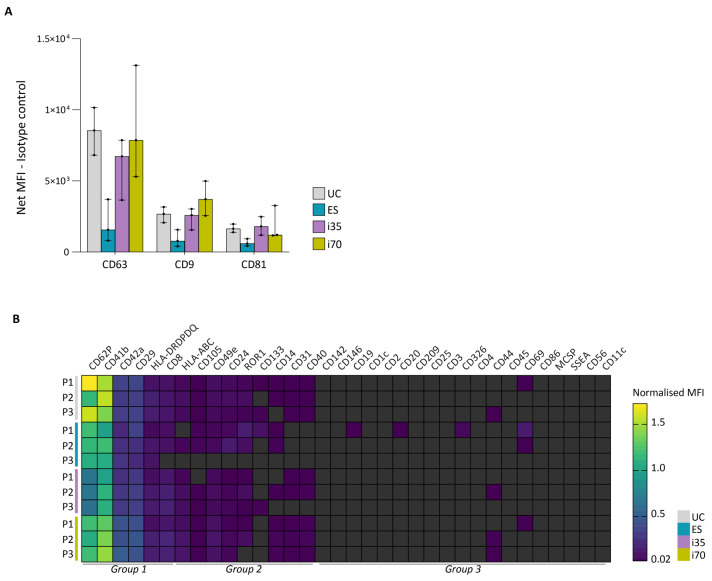
EV surface phenotyping. (**A**) Abundance of the three tetraspanins measured by the kit (i.e., CD63, CD9, and CD81) expressed as net median fluorescence intensity (MFI) minus the MFI measured for the isotype control. Bars represent the median with range. (**B**) Normalised MFI for the different markers targeted by the kit. Markers were grouped (group 1–3) based on their level of expression, as described in the method section. Dark grey cells: normalised MFI < 0.02.

The relative abundance of 37 surface epitopes was assessed using the commercial MACSPlex Exosome kit, human (Figure 4 and Supplementary File S1). We first evaluated the signal intensities of the three tetraspanins targeted by the kit—CD63, CD9, and CD81. Consistent with WB results, the tetraspanin signals were lower in ES-EVs compared to the other preparations. Both i35- and i70-EVs displayed high tetraspanin levels, although CD63 detection in i70-EVs showed high variability (Figure 4A). In contrast to WB, CD81 and CD63 signals in UC-EVs were comparable to those in i35- and i70-EVs. This discrepancy may be attributed to methodological differences, since the MACSPlex assay was performed using an equal number of EVs (as determined by NTA), whereas WB was based on equal sample volumes, with subsequent signal normalisation to total protein load.

The relative abundance of the remaining 34 surface epitopes was determined by normalising the mean fluorescence intensity (MFI) of each marker to that of tetraspanins, which were used as detection reagents. Fifteen epitopes were reliably detected in our samples, six of which had high normalised MFI (i.e., CD62P, CD41b, CD42a, CD29, HLA-DRDPDQ, CD8) and nine with medium intensity (i.e., HLA-ABC, CD105, CD49e, CD24, ROR1, CD133, CD14, CD31, CD40). These epitopes encompass markers of platelet, leukocyte (including monocytes, neutrophils, B, T, and natural killer cells), and endothelial origin, all commonly present in circulating body fluids.

EVs enriched with the different methods exhibited similar profiles in terms of normalised MFI, particularly for group 1 markers (Figure 4B). Although group 2 markers were detected with medium intensity in all samples, their signals showed greater variability across methods, with ES-EVs showing the lowest overall intensity.

### 2.4. Different Enrichment Methods Are Associated with Variations in EV Protein Cargo

**Figure 5:**
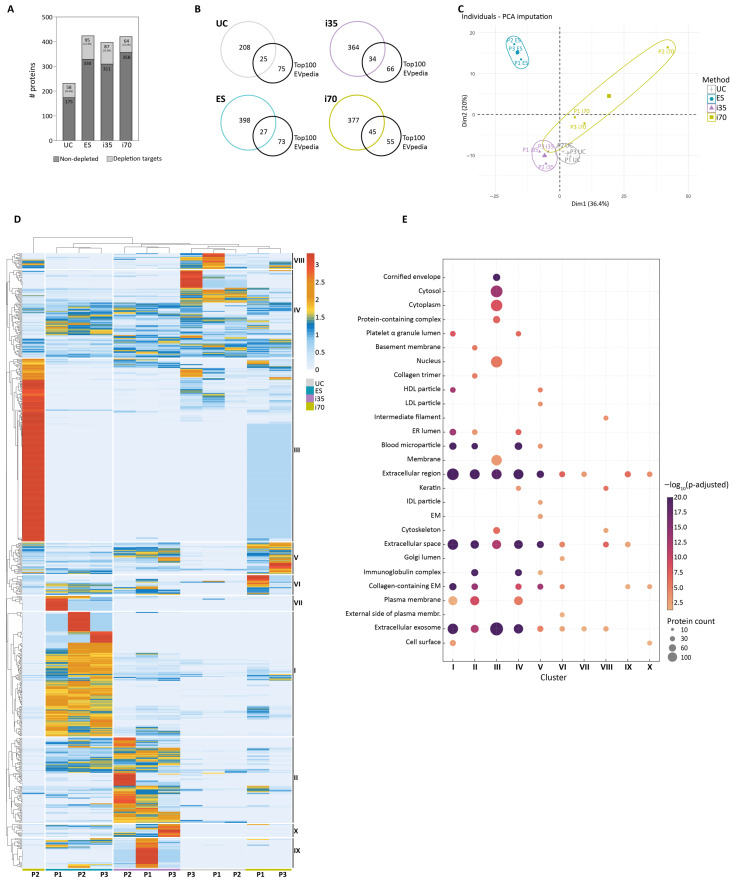
Proteomics results. (**A**) Number of proteins identified in the different EV preparations. Numbers on the charts represent identified proteins targeted by the immunodepletion column (light grey) and other proteins identified (dark grey). (**B**) Venn diagrams showing the proportion of the top 100 EV proteins enlisted by EVpedia (https://evpedia.info/evpedia2_xe/, accessed on 26 May 2025) detected in the different EV preparations. (**C**) Principal component analysis (PCA) was performed on the proteins identified by mass spectrometry. (**D**) Hierarchical Clustering Analysis (HCA) of the protein normalised abundances and (**E**) GO enrichment analysis for the Cellular Component terms (CC) performed on the 10 clusters identified in (**D**). HDL: high-density lipoproteins; LDL: low-density lipoproteins; ER lumen: endoplasmic reticulum lumen; IDL: intermediate-density lipoproteins; EM: extracellular matrix.

EVs were analysed by LC-MS/MS after immunodepletion of the 14 most abundant serum proteins. Overall, 651 proteins were identified across the different samples (Supplementary File S1). The ES and i70 methods yielded the highest number of identified proteins, i.e., 425 and 422, respectively. Similarly, 398 proteins were identified in i35-EVs, while a lower number of proteins was identified in UC-EVs, i.e., 233 (Figure 5A). Interestingly, 19.7% of all identified proteins were unique to i70-EVs, while 12.3% where exclusive to ES-EVs (Supplementary Figure S3A). To further investigate the protein cargo of the different preparations, we considered (i) the identification of proteins targeted by the immunodepletion and (ii) the identification of common EV-associated proteins. As immunodepletion reduces but does not eliminate target proteins, a higher carryover of serum proteins during EV enrichment likely results in a less efficient immunodepletion. Consistent with this hypothesis, UC- and ES-EVs displayed the largest proportions of immunodepletion targets (24.9% and 22.4%, respectively), whereas i70-EVs displayed the least (15.2%) (Figure 5A). UC-EVs were particularly rich in albumin, while samples from the other methods were more affected by lipoprotein contaminations (Supplementary Figure S3B), as observed by WB.

To evaluate whether the enrichment methods differed in their ability to identify proteins commonly associated with EVs, we compared our datasets with the top 100 EV proteins enlisted in EVpedia as the most frequently identified within human EV proteomes, thus considered more conserved across matrices (https://evpedia.info/evpedia2_xe/, accessed on 26 May 2025) [30]. The i70 method yielded the highest proportion of top 100 proteins (45%), while UC yielded the lowest (25%) (Figure 5B).

Principal Component Analysis (PCA) showed that EV proteomes clustered primarily according to the enrichment method rather than the biological sample, except for i70, supporting the influence of enrichment strategy on downstream protein identifications (Figure 5C). To further explore method-dependent differences, Hierarchical Clustering Analysis (HCA) was performed, followed by gene ontology (GO) enrichment analysis of cellular component (CC) terms on the clusters (Figure 5D,E, and Supplementary File S1). Except for i70 P2, samples processed with the same method clustered together (Figure 5D), confirming consistent protein identification across biological samples, as also indicated by PCA. All methods identified vesicle-related proteins, since the GO terms “Extracellular exosome”, “Extracellular region”, and “Extracellular space” were enriched in most clusters (Figure 5E). Overall, i35- and UC-EVs were enriched in immunoglobulins and extracellular matrix-associated proteins, whereas proteins associated with sub-cellular organelles, including Golgi, endoplasmic reticulum, and nucleus, were identified across the different methods, particularly in i70-EVs.

Cluster I, predominantly enriched in ES-EVs, encompassed 134 proteins, of which only two—gelsolin and PDCD6 (Programmed cell death protein 6)—were enlisted among the top 100 EVpedia proteins. Notably, PDCD6—through the interaction with programmed cell death 6-interacting protein (PDCD6IP)—is involved in the formation of the endosomal sorting complexes required for transport (ESCRT) [31].

Similarly, cluster II, comprising 92 proteins, was predominantly enriched in i35-EVs and included only five top 100 proteins, among which syntenin-1 is known as implicated in vesicle biogenesis and trafficking [32].

Cluster III was the largest, containing 197 proteins. It was mostly represented in i70-P2-EVs and enriched in cytosolic proteins. At this stage, it is difficult to establish whether the different proteome profile of P2-i70 is due to technical or biological variability associated with the carryover of platelet and red blood cells in the P2 sample (Supplementary File S2). Cluster III included the largest number of top 100 proteins (32 proteins), encompassing structural proteins such as annexin A1 (ANXA1) and annexin A2 (ANXA2), as well as proteins involved in EV biogenesis and trafficking, including clathrin heavy chain 1 (CLH), transitional endoplasmic reticulum ATPase (TERA), and Ras-related protein Rab-10 (RAB10) [33,34,35].

Cluster IV (94 proteins), common to all EV preparations, encompassed numerous serum proteins, including seven complement components or associated proteins (i.e., C4BPA, C4BPB, C1QA, C1QC, C1R, C1S, and CO3), 33 immunoglobulin chains, and other abundant serum proteins (e.g., serotransferrin, protease inhibitors, albumin, beta-2 macroglobulin, fibrinogen).

Cluster V (34 proteins), predominantly associated with qEV1-EVs, was enriched in GO terms related to HDL and LDL particles, since it included six apolipoproteins.

Clusters from VI to X contained fewer proteins, spanning from 32 identifications for cluster IX to 14 for cluster X. Cluster VI (21 proteins) and cluster VII (16 proteins) were primarily enriched in qEV1- and/or ES-EVs. Cluster VIII (17 proteins), enriched in UC-EVs and i70-EVs, included cytoskeletal proteins and keratins, possibly representing contamination from serum sample collection or preparation. Clusters IX and X (32 and 14 proteins, respectively) were mostly represented in ES- and i35-EVs proteomes.

### 2.5. miRNA Signatures Are Partially Influenced by the EV Enrichment Method

miRNA profiling by NGS was performed on EVs enriched using 5 different methods, since ExoRNeasy (ER) was included specifically for this task.

Analysis of total RNA yield and integrity showed that UC, ES, and ER preparations were enriched in small RNAs with well-defined profiles, typical of EV-associated RNA cargo (Supplementary Figure S4). Conversely, i35- and i70-EVs displayed flattened and less defined profiles, indicating lower RNA yields and a possible lack of enrichment in small RNA species. This observation was confirmed by total RNA quantification, showing the highest quantities in UC-, ES-, and ER-EVs, while i35- and i70-EVs yielded less RNA.

Sequencing metrics for each isolation method are summarised in Table 1, providing a quantitative framework that supports the downstream analysis of RNA yield and biotype distribution. One ER sample (ER-P3) was excluded from further analysis because of the low number of sequenced reads compared to the other two ER samples.

**Figure 6:**
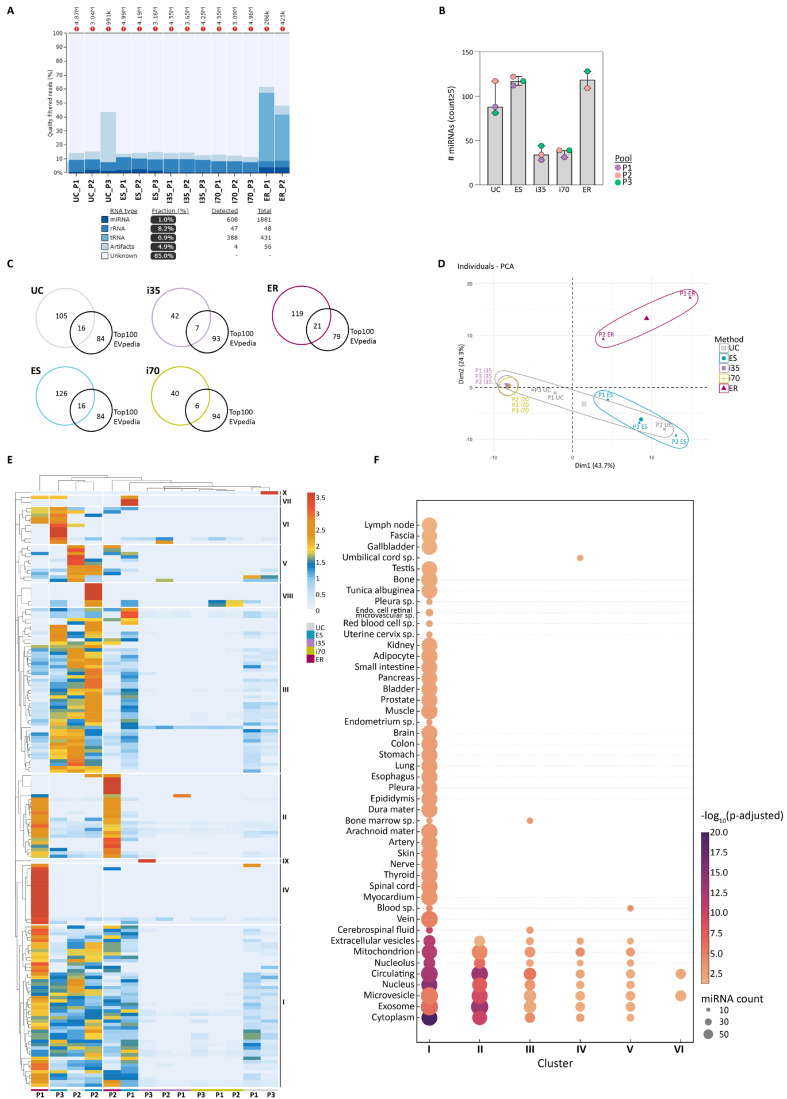
RNA sequencing results. (**A**) Taxonomic classification of RNA biotypes across the different EV preparations. Bar plots indicate the relative proportion of RNA types identified per sample, including miRNAs, rRNAs, and tRNAs. (**B**) Number of miRNAs identified in each sample (median and range). (**C**) Venn diagrams showing the proportion of the top 100 EV miRNAs enlisted by EVpedia (https://evpedia.info/evpedia2_xe/, accessed on 26 May 2025) detected in the different EV preparations. (**D**) Principal component analysis (PCA) was performed on the miRNA sequences detected by NGS with counts ≥ 5. (**E**) Hierarchical Clustering Analysis (HCA) of miRNA expression profiles. (**F**) GO enrichment analysis of clustered miRNA based on cellular component ontology using ORA. The dot plot shows significantly enriched cellular component terms identified by miEEA. Sp: specific.

EV RNA composition and biotype distribution were further assessed using the miRTrace v1.0.1 tool. UC- and ES-EVs showed a relatively balanced distribution of miRNAs and rRNAs, consistent with a successful enrichment of canonical EV RNA species (Figure 6A). In contrast, i35- and i70-EVs displayed a dominant presence of rRNA and limited miRNA content, possibly reflecting residual ribonucleoproteins or suboptimal EV recovery. Interestingly, ER-EVs displayed a distinct profile, with the highest relative abundance of miRNAs and rRNAs, and uniquely, the presence of tRNAs. These data suggest that ER may provide a broader RNA repertoire, capturing additional RNA species potentially missed by other isolation strategies.

To evaluate miRNA diversity, the number of unique human miRNAs detected post-sequencing (counts ≥ 5) was quantified (Supplementary File S3). ES-EVs yielded the highest number of distinct miRNAs (142 miRNAs identified in at least one of the three biological samples), followed by ER- (140 miRNAs) and UC-EVs (121 miRNAs) (Figure 6B). i35- and i70-EVs identified only 49 and 46 miRNAs, respectively. These results reinforce previous findings from TapeStation and taxonomic profiling, indicating that qEV-based methods may be less efficient in capturing a broader spectrum of small RNA species carried by stored serum EVs. Across all EV enrichment methods, a core of 25 miRNAs was consistently detected in all samples. These included broadly expressed miRNAs, such as let-7a-5p, let-7b-5p, let-7f-5p, miR-16-5p, miR-93-5p, miR-191-5p, and miR-3184-3p. The full list is provided in Supplementary File S3.

As performed for proteins, we evaluated the proportion of EVpedia Top 100 miRNA (https://evpedia.info/evpedia2_xe/, accessed on 26 May 2025) detected across the different preparations. ER-EVs contained 21% of the top 100 miRNAs, followed by UC- and ES-EVs (16% each), while i35- and i70-EVs included only 7% and 6%, respectively (Figure 6C).

PCA showed that EV miRNA only partially clustered according to the EV enrichment methods (Figure 6D), even though the two ER-EVs preparations clustered together, as well as qEV1-EVs, UC-P2-EVs grouped with ES-EVs, and UC-P1/P3-EVs were closer to qEV1-EVs.

HCA identified ten distinct miRNA expression clusters, indicating that the method of EV isolation partially influences miRNA cargo characterisation (Figure 6E). Clusters II and IV were predominantly associated with ER-EVs, suggesting that this method may efficiently capture small RNA not recovered by the other approaches. ER-EVs showed similar expression profiles across multiple clusters, reflecting high reproducibility and a stable miRNA signature.

Clusters I and III, the most represented, included broadly expressed miRNAs such as miR-223-3p, let-7a-5p, and miR-30a-3p [36,37], which were present across multiple methods (UC, ES, ER) and frequently reported as abundant in EVs [38,39].

Consistent with PCA and in contrast to proteomics results, UC-, ES-, and ER-EVs did not clearly cluster by enrichment method or biological samples. This could be due to the smaller number of detected miRNA sequences compared to the number of proteins. In contrast, i35- and i70-EVs showed greater heterogeneity and lower consistency in miRNA content, as reflected in clusters IV and V.

To explore the cellular origin of the identified miRNAs, GO enrichment analysis was performed using the miEEA tool [40], focusing on the CC category through Over-Representation Analysis (ORA) (Figure 6F). Cluster I exhibited the broadest and most diverse enrichment, with significant representation of EV-associated terms (extracellular vesicle, exosome, microvesicles, and circulating component), suggesting these miRNAs originate from multiple cell types and may contribute to intercellular communication via various EV populations. Cluster II was strongly enriched in EV-related components, particularly for the nucleus and nucleolus, with high statistical significance. Clusters III to VI showed limited GO enrichment, likely reflecting lower specificity.

## 3. Discussion

In the present study, we demonstrated that EVs can be efficiently enriched from as little as 250 μL of biobanked serum for pre-clinical research purposes. Our comprehensive evaluation of the enriched vesicular populations revealed that the choice of the enrichment method influences downstream analyses, particularly the characterisation of EV molecular cargo. The main advantages and limitations of each method investigated are summarised in Table 2, along with considerations of costs, time of execution, and ease of use.

Overall, all methods tested in our study—UC, SEC-based methods, and a membrane-based affinity binding method—allowed enriching small EVs, consistent with previous reports [24,41].

The particle yield was comparable among UC, i35, and i70, whereas ES enriched a higher number of EVs but also a higher amount of contaminating proteins, resulting in an overall purity similar to that of the other preparations. Indeed, since NTA cannot distinguish EVs from lipoproteins, a higher particle count does not necessarily correspond to a larger number of EVs.

According to a recent survey among EV researchers, differential ultracentrifugation remains the most widely used EV enrichment technique, even though SEC-based methods are increasingly adopted, probably due to their ability to preserve EV integrity [14]. In our study, UC enriched a broader EV population compared to SEC-based methods, likely reflecting its tendency to induce vesicle aggregation and structural alterations. Moreover, UC efficiency can be influenced by sample viscosity, leading to inter-sample variability and co-enrichment of contaminants [26,42,43]. Blood-derived EV preparations are, in fact, prone to contamination by soluble proteins, like albumin, or co-enrichment of lipoprotein particles [24,44], the level of which might depend on the EV enrichment strategy, as also observed here. Although UC may co-enrich lipoprotein particles or other soluble contaminants, this does not substantially affect the overall interpretation of NGS data, as miRNAs detected in EVs are considered more stable, and potentially more abundant, compared to those freely circulating [45]. Accordingly, the shared miRNA subset identified across all enrichment methods likely reflects a robust EV-associated signature. Therefore, the choice of EV enrichment strategy should be guided by the type of sample to be analysed, the volume available, and intended downstream analyses, as both yield and purity can affect the reliability of EV-based biomarker discovery [26].

In our study, UC and qEV1 provided consistent surface phenotyping results, whereas ES-derived EVs displayed lower signal intensities. The most efficient methods for downstream proteomic analyses, in terms of number of identified proteins, were i70 nm and ES, although the latter exhibited high carryover of soluble serum proteins. Interestingly, i70 showed high variability in the number of identified proteins across the three biological samples, raising the question of whether this method might be better suited to capturing the intrinsic variability that characterises biological specimens. However, these methods were affected by either high variability between biological samples or high carryover of soluble serum proteins, respectively. Based on protein identifications, SEC-based methods predominantly co-enriched lipoproteins, while UC preparations were more contaminated with albumin. Through the introduction of the immunodepletion step, we improved the number of identified proteins compared to other studies [41], while reducing the starting volume of serum.

UC, ES, and ER were all associated with the identification of an adequate number of vesicular miRNAs, with ER achieving the highest proportion of EV-associated miRNAs. In this regard, it should be noted that the EVpedia Top 100 list includes proteomes and miRNomes from all sources of EVs, not exclusively from serum-derived EVs. Additionally, only two of the three biological samples were analysed using ER, likely reducing the number of total identifications. In contrast, qEV1 preparations produced very low RNA yields and were therefore not appropriate for miRNA biomarker investigations.

Despite some controversy, serum is now recognised as a suitable matrix for EV-based biomarker research [44,46,47]. Our results show that, also when biobanked and available in limited volume, serum can be efficiently used for various downstream EV investigations. Nonetheless, some drawbacks associated with the use of this type of sample should be taken into account. In particular, platelet-derived EVs can be released in serum ex vivo during the activation of the blood coagulation cascade [48]. For instance, a comparative study has reported higher concentrations of CD9^+^-EVs and other platelet-derived EVs in serum than in paired plasma samples or platelet-poor plasma [19], although opposite results have also been described, showing increased CD9^+^ and CD41a^+^ platelet-derived EVs in EDTA plasma [44]. In our study, regardless of the enrichment method, CD63 was the predominant surface marker, while CD9 was less abundant, in agreement with previous reports [44]. Additionally, seven platelet-associated markers, i.e., CD41b, CD42a, CD62P, CD29, and CD31, were also detected in our serum-derived EVs, though these are not exclusive to platelets, as they are also expressed by other cell types [49]. The proper enrichment of serum-derived EVs was further supported by CD81^+^-EVs, known to be absent in platelet-derived EVs [19], which showed a profile similar to CD9 and CD63.

Serum also presents some advantages. Its preparation is more standardised compared to plasma, which instead can be collected using different anticoagulants and centrifugation protocols [50,51]. Furthermore, it is less affected by residual cells, as they are trapped in the clot during separation [19].

In the present study, UC provided very good results, except for the analysis of the protein cargo, where high albumin contamination was observed. In our laboratory, we have previously observed significant operator-dependent variability in UC performance; however, this was not evident here since all samples were processed by the same operator. Future studies should address operator effects across different enrichment methods, as they might influence reproducibility and the clinical translation of EVs research.

Overall, we detected a lower number of proteins and miRNAs by multi-omics compared with other published studies [52,53]. This is likely due to the reduced volume of starting material, which aligns with the constraints of using biobanked samples. Nonetheless, because abundant biomarkers are generally the most amenable to translation into rapid tests, the number of identifications achieved here is suitable for biomarker discovery.

Here, we only evaluated individual approaches for EV enrichment, although previous reports suggest that combining UC and SEC can improve EV purity while preserving functional properties compared to either method alone [24,41]. In our study, we did not evaluate EV functional properties but rather the possibility of using biobanked serum for biomarker discovery, where limited sample volume is often a constraint. Since combining methods typically enhances purity at the cost of lower yield, such an approach might not be optimal when the sample quantity is limited.

Finally, for technical feasibility, we analysed pooled samples. Nonetheless, all experimental procedures were performed using 250 μL of starting material, supporting the translatability to individual biobanked samples.

Our results thus indicate that biobanked serum is a valuable source for EV-based pre-clinical research, although caution should be taken when interpreting data on platelet-derived EVs. Through a comprehensive comparison, we provide experimental evidence to support method selection for the most common EV downstream analyses. In the context of biomarker studies, all methods examined—except for ES—proved suitable for EV surface phenotyping. Regarding multi-omics characterisation of EV molecular cargo, our data highlight that no single enrichment method is optimal for both proteomics and miRNomics purposes. Nonetheless, ES might offer a good compromise in terms of protein and miRNA identification, while also being highly scalable, thereby reducing the time of sample processing for large cohort studies. When considered individually, ER was the most suitable method for miRNA profiling, while i70 yielded the highest number of vesicular proteins, although its scalability depends on the availability of automatic collection systems. As reported in the literature [53], and as observed here, different methods might lead to different degrees of purity of the enriched vesicle populations. However, depending on the study design, impurities might have little effect on the downstream analyses. In the specific case of serum-derived EVs, contamination with circulating proteins may significantly impact protein identification but not miRNA detection, as observed for UC-EVs [54].

In conclusion, our findings underscore the need to consider study objectives, cohort size, and the platforms available for experimental procedures when selecting EV enrichment strategies for biomarker research. Indeed, the choice of EV isolation method substantially affects the detection of miRNA and protein cargo, with potential implications for biological interpretation and biomarker discovery. Additionally, EV-based biomarker discovery should also include verification or validation steps to assess the reliability of proposed markers and to advance EV research towards clinical applications.

## 4. Materials and Methods

### 4.1. Study Design, Sample Selection, and Pooling

Three pools of five biobanked serum samples from age- and sex-matched healthy subjects were prepared by combining equal volumes of serum from each donor and divided into 250 μL aliquots (Figure 1). Serum samples were retrieved from the Tropica Biobank (BBMRI-eric ID: IT_1605519998080235) of the IRCCS Sacro Cuore Don Calabria Hospital and anonymised before use. The study is covered by the Protocol n° 63961/2022, approved by the Ethical Committee of Verona and Rovigo provinces on 19 October 2022. All subjects have signed an informed consent. The study was performed in accordance with the Declaration of Helsinki.

The samples analysed are representative of serum samples stored within the Tropica Biobank, since they followed the same collection, preparation, and storage procedures. Briefly, samples were drawn using a winged butterfly needle (21 gauge) with vacuum blood collection tubes (CAT Serum Separator Clot Activator Tube, Greiner Bio-One, Kremsmünster, Austria), transported, and stored upright vertically at room temperature until processing. Serum was prepared within 8 h from collection by centrifugation at 1650× *g* for 8 min at room temperature, and then separated into 200–500 µL aliquots in 1.5 mL microtubes (Sarstedt, Nümbrecht, Germany) and stored at −80 °C. At the visual inspection, none of the included samples presented signs of haemolysis or hyperlipidaemia. Details of the pre-analytical processing and quality check of the pooled samples employed in the present study are reported through the Minimal Information for Blood EV research (Supplementary File S2 and Supplementary Figure S1) [17].

Each pooled serum (250 μL) underwent EV enrichment using ultracentrifugation, Exo-spin^TM^ (Cell Guidance Systems, Cambridge, UK), Izon qEV1-35 nm and qEV1-70 nm columns (Izon Sciences, Christchurch, New Zealand), and was submitted to the following downstream analyses: transmission electron microscopy (TEM), nanoparticle tracking analysis (NTA), Western blot (WB), surface phenotyping, and proteomics. For the determination of the miRNA cargo, the ExoRNeasy (Qiagen, Hilden, Germany) enrichment method was added to those mentioned above.

### 4.2. EVs Enrichment

Pooled serum samples (250 μL) were first centrifuged at 2000× *g* for 30 min to remove larger debris, followed by another centrifugation at 10,000× *g* for 30 min to remove larger particles, prior to enrichment with the different methods as described hereafter.

#### 4.2.1. Ultracentrifugation (UC)

Samples were diluted 1:5 with 0.22 μm filtered PBS and ultra-centrifuged at 100,000× *g* for 2 h at +4 °C using a Fiberlite F50L-24 × 1.5 Fixed-Angle Rotor on a Sorvall^TM^ WX ultracentrifuge (Thermo Scientific, Waltham, MA, USA). EV pellets were washed with 0.22 μm filtered PBS at 100,000× *g* for 2 h at +4 °C. Washed pellets were re-suspended in 50 μL of PBS and filtered through 0.22 μm spin filters (Corning, Corning, NY, USA).

#### 4.2.2. Exo-Spin^TM^ Mini Blood Columns (ES)

ES columns, which should enrich particles between 30 and 200 nm, were employed according to the manufacturer’s instructions. Briefly, EVs were first precipitated with Exo-spin buffer for 1 h at +4 °C prior to centrifugation at 16,000× *g* for 30 min. EV pellet was then warmed at 37 °C for 10min, re-suspended in PBS, and purified through the Exo-spin^TM^ column. EV preparations were then concentrated with Amicon^®^ Ultra Centrifugal Filter, 10 kDa MWCO (Millipore, Burlington, MA, USA) to a final volume of 50 μL.

#### 4.2.3. Izon qEV1 Columns

Both qEV1 35nm (i35, particle size range of 35–350 nm) and qEV1 70 nm (i70, particle size range of 70–1000 nm) were employed according to manufacturers’ instructions. EVs were eluted using filtered PBS and collected manually as a unique fraction (4 fractions of 0.7 mL each). Following Izon separation, samples were concentrated with Amicon^®^ Ultra Centrifugal Filter, 10 kDa MWCO (Millipore) to a final volume of 200 μL.

After enrichment, the protein concentration of all samples (UC, ES, i35, i70) was measured by Qubit protein assay (ThermoFisher Scientific). Samples were used immediately or stored overnight at +4 °C for further use.

#### 4.2.4. ExoRNeasy (ER)

ER was used to purify vesicular RNA directly from 250 µL of pooled serum, following the manufacturer’s instructions. Briefly, samples were diluted with buffer XBP and bound to the column. After washing, vesicles were lysed and eluted with QIAzol reagent. Chloroform was added to the lysate and centrifuged. The RNA present in the aqueous phase was collected and extracted using the RNeasy MinElute spin column. RNA was eluted in 14 µL of RNase-free water.

### 4.3. Transmission Electron Microscopy

Freshly prepared EVs from pool#1 were visualised by transmission electron microscopy (TEM). Briefly, one drop of sample solution (approximately 25 µL) was placed on a 400-mesh holey film grid and stained with 2% uranyl acetate for 2 min. The sample was observed with a Tecnai G2 (FEI) transmission electron microscope operating at 120 kV. Images were captured with a Veleta (Olympus Soft Imaging System, Münster, Germany) digital camera.

### 4.4. Nanoparticle Tracking Analysis

Particle size and concentration were analysed by Nanoparticle Tracking Analysis (NTA) using a NanoSight NS300 instrument (Malvern Panalytical, Malvern, UK) equipped with a 488 nm laser and a scientific CMOS camera. EV preparations were vortexed and diluted (1:100–1:1000) in order to obtain a concentration and particles/frame within the recommended measurement range. Three consecutive videos of 60 s per sample were acquired with a syringe pump speed set to 20 and subsequently analysed using the NTA 3.4 software (Malvern).

### 4.5. Western Blot

Samples were dried under vacuum and re-suspended in 50 μL of PhophoSafe^TM^ Extraction Reagent (Merck Millipore, Burlington, MA, USA) to extract EV proteins. Six μL of each sample were separated on a Mini-PROTEAN^®^ TGX^TM^ precast 4–20% gel (Bio-Rad, Hercules, CA, USA) and transferred onto a nitrocellulose membrane.

The following antibodies were used under non-reducing conditions using 1x Laemmli protein sample buffer for SDS-PAGE (Bio-Rad): mouse monoclonal anti-human CD81 antibody (clone M38, 2 μg/mL, Invitrogen) and mouse monoclonal anti-human CD63 antibody (clone TS63, 1 μg/mL, Abcam, Cambridge, UK), both detected with polyclonal goat anti-mouse immunoglobulins/HRP (horseradish peroxidase) (Dako, Glostrup, Denmark).

The following antibodies were used under reducing conditions using 1x Laemmli protein sample buffer for SDS-PAGE (Bio-Rad) with 355 mM 2-Mercaptoethanol: rabbit monoclonal anti-human flotillin-1 (clone EPR6041, 1 μg/mL, Abcam) and rabbit polyclonal anti-human apolipoprotein AI (0.2 μg/mL, Abcam), both detected with polyclonal goat anti-rabbit immunoglobulins/HRP (Dako); mouse monoclonal anti-human calnexin (CANX, clone CANX/1543, 1 μg/mL, Abcam) detected with polyclonal goat anti-mouse immunoglobulins/HRP (Dako).

The chemiluminescent signal was detected after membrane exposure to Clarity Max Western ECL Substrate (Bio-Rad) on a ChemiDoc Imaging System (Bio-Rad).

Caco-2 cell lysate was used as a positive control for anti-Flot-1, anti-CANX, anti-CD81, and anti-CD63 antibodies, while human serum was used as a positive control for anti-APO-AI antibody.

Band quantification was performed with Image Lab^TM^ software (v6.0.1) (Bio-Rad), using the stain-free membrane signal to compute the normalisation factor and obtain the normalised band volume.

### 4.6. EVs Surface Phenotyping

EVs’ surface phenotyping was determined using the MACSPlex Exosome kit, human (Miltenyi Biotec). The assay was performed according to the manufacturer’s instructions using 10^9^ EVs per sample, as established by NTA, and detection with a cocktail comprising CD9, CD63, and CD81 detection reagents. Data were acquired on a CytoFlex flow cytometer (Beckman Coulter, Brea, CA, USA). To establish the relative abundance of the different surface markers, raw data were analysed as reported by Ekström and colleagues with slight modifications [55]. Briefly, scatter plots were first visually inspected to verify the signal in the isotype control regions. Each MFI was then subtracted from the blank value and from the signal registered in the isotype control region. To obtain normalised MFI values, data were finally divided by the mean of tetraspanin signals (i.e., CD63, CD81, and CD9). All EV sub-populations having 75% of the normalised MFI values across the entire population below a 0.02 threshold were considered as not detected (group 3). EV sub-populations having normalised MFI between 0.02 and 0.1 in 75% of the samples were considered as detected with medium/low intensity (group 2), while samples having normalised MFI equal to or above 0.1 in 75% of the samples were detected with high intensity (group 1).

### 4.7. Protein Identification and Quantification by LC-MS/MS

EV samples were dried under vacuum and lysed in 50 μL of 0.1% RapiGestSF surfactant (Waters Corporation, MA, USA) in 0.1 M triethylammonium bicarbonate buffer (TEAB), pH 8.0. Samples were vortexed, heated at 80 °C for 10 min, and sonicated before centrifugation at 14,000× g for 10 min at +4 °C. The supernatant containing EV proteins was collected and depleted of the 14 most abundant serum proteins (i.e., α1-acid glycoprotein, α1-antitrypsin, α2-macroglobulin, albumin, apolipoprotein AI, fibrinogen, haptoglobin, IgA, IgD, IgE, IgG, IgG light chains, IgM, and transferrin) using High-Select^TM^ Top14 Abundant Protein Depletion Resin (ThermoFisher Scientific), following the manufacturer’s instructions. Depleted samples were then buffer-exchanged and concentrated using Amicon Ultra-0.5 centrifugal filter devices (3 kDa cut-off) (Millipore), and protein concentration was measured using Qubit Protein Assay (ThermoFisher Scientific).

Proteins were then reduced in 25 µL of 100 mM NH4HCO3 with 2.5 μL of 200 mM dithiothreitol (DTT) (Merck) at 60 °C for 45 min, and next alkylated with 10 μL 200 mM iodoacetamide (Merck) for 1 h at RT in the dark. Iodoacetamide excess was removed by the addition of 200 mM DTT. Proteins were then digested with trypsin. The digests were dried under vacuum and then desalted. Digested peptides were analysed on an Ultimate 3000 RSLCnano coupled directly to an Orbitrap Exploris 480 with a High-Field Asymmetric Waveform Ion Mobility Spectrometry System (FAIMS) (all Thermo Fisher Scientific). Samples were injected onto a reversed-phase C18 column (15 cm × 75 µm i.d., Thermo Fisher Scientific) and eluted with a gradient of 6% to 95% mobile phase B over 41 min by applying a flow rate of 500 nL/min, followed by an equilibration with 6% mobile phase B for 1 min. The acquisition time of one sample was 41 min, and the total recording of the MS spectra was carried out in positive resolution with a high voltage of 2500 V and the FAIMS interface in standard resolution, with a CV of −45 V. The acquisition was performed in data-independent mode (DIA): precursor mass range was set between 400 and 900, isolation window of 8 *m*/*z*, window overlap of 1 *m*/*z*, HCD collision energy of 27%, orbitrap resolution of 30,000, and RF Lens at 50%. The normalised AGC target was set to 1000, the maximum injection time was 25 ms, and the microscan was 1. For DIA data processing, DIA-NN (version 1.8.1) was used: the identification and quantification were performed with “library-free search” and “deep learning-based spectra, RTs and IMs prediction” enabled; database: human-reviewed (Uniprot, downloaded on 20 March 2025). Enzyme was set to Trypsin/P, precursors of charge state 1–4, peptide lengths 7–30, and precursor *m*/*z* 400–900 were considered with a maximum of two missed cleavages. Carbamidomethylation on C was set as a fixed modification, and Oxidation on M was set as a variable modification, using a maximum of two variable modifications per peptide. FDR was set to 1%.

Principal component analysis (PCA) was carried out in R (version 4.5.0) on the dataset containing normalised protein intensities. The package factoextra (available at: https://CRAN.R-project.org/package=factoextra, accessed on 26 May 2025) was used to visualise the plot of individuals within the principal component space (dimensions 1 and 2).

Hierarchical cluster analysis (HCA) was conducted on the scaled proteins dataset in R, with clusters determined using complete linkage clustering and Euclidean distance matrix. The heatmap was generated using the pheatmap package (available at: https://CRAN.R-project.org/package=pheatmap, accessed on 26 May 2025). Protein GO annotation was performed on the first ten clusters of proteins using David Functional Annotation Tool [56,57]. For each cluster, cellular component (CC) GO terms significantly represented across the dataset were considered (Bonferroni-adjusted *p*-value <0.05). The top 10 most represented terms (i.e., percentage of detected genes/total genes) were considered as enriched within each cluster and were visualised as dot plots using Python 3.11.

### 4.8. miRNA Identification by Next-Generation Sequencing

#### 4.8.1. RNA Extraction, miRNA Library Preparation, and Sequencing

Total RNA from EVs prepared using UC, ES, i35, and i70 was extracted using the miRNeasy kit (Qiagen), according to the manufacturer’s instructions. Samples prepared with the ExoRNeasy kit (ER) (Qiagen) were ready to use, since the kit directly purifies vesicular RNA.

For all samples, RNA concentration and quality were assessed with the Qubit^TM^ 4 Fluorometer using the Qubit RNA HS Assay Kit (ThermoFisher Scientific) and the 4200 TapeStation System with the High Sensitivity RNA ScreenTape (Agilent, Santa Clara, CA, USA).

For miRNA profiling, 5 µL of RNA from all the isolation methods were processed using the QIAseq miRNA Library Kit (Qiagen). Library quality controls were performed with Qubit^TM^ 4 Fluorometer (Qubit RNA HS Assay Kit, ThermoFisher Scientific) and the 4200 TapeStation System using the High Sensitivity D1000 screen tape (Agilent). Libraries were sequenced on an Illumina NextSeq1000 sequencer (Illumina, San Diego, CA, USA).

#### 4.8.2. Bioinformatics Analysis

Reads were quality-assessed using the FastQC tool, v.0.12.1. Adapter trimming and quality filtering were performed using the Cutadapt tool, v. 5.0. Unique Molecular Identifiers (UMIs) were extracted from raw FASTQ reads with UMI-tools v1.1.6, using the following parameters: --extract-method=regex, --bc-pattern=‘.+(?P<discard_1>AACTGTAGGCACCATCAAT){s<=2}(?P<umi_1>. [36])(?P<discard_2>.+)’). Additional quality control metrics were evaluated with miRTrace v1.0.1. Processed reads were then aligned to the human reference genome (hg38) using Bowtie v1.3.1. UMI-based deduplication was performed with the dedup function in UMI-tools, and miRNAs quantification was performed using bedtools coverage-counts v2.31.1. Only miRNAs with at least 5 raw reads for each individual sample were considered for further analyses.

PCA and HCA analyses were performed as reported above for proteins, using the miRNA counts dataset and the scaled miRNA dataset, respectively.

Gene Ontology (GO) enrichment analysis was performed using the miEEA tool [40] (microRNA Enrichment Analysis and Annotation), applying Over-Representation Analysis (ORA) within the Cellular Component category. Only terms with a Benjamini–Hochberg adjusted *p*-value < 0.05 were considered. GO enrichment results were visualised as dot plots using Python 3.11.

## Figures and Tables

**Table 1:** Statistics of the internal analysis of NGS results.

Sample	# Fragments	# Fragments After QC Control	# Fragments After Trimming *	# Mapped Fragments (%)	# Mapped After UMI Dedup.
UC_P1	8,859,531	8,000,108	5,258,922	2,668,369 (50.74%)	1,805,035
UC_P2	6,339,286	5,813,760	3,365,675	1,819,089 (54.05%)	1,371,515
UC_P3	2,981,507	2,599,145	984,244	721,152 (73.27%)	539,793
ES_P1	9,292,803	8,585,735	5,703,679	3,078,327 (53.97%)	1,881,529
ES_P2	8,656,862	7,980,448	4,738,364	2,604,078 (54.96%)	1,716,147
ES_P3	7,269,556	6,792,316	3,582,768	1,973,226 (55.08%)	1,565,703
i35_P1	8,039,458	7,055,985	4,664,841	2,343,190 (50.23%)	1,017,582
i35_P2	6,389,430	5,660,226	3,724,139	1,807,577 (48.54%)	1,073,088
i35_P3	7,388,006	6,486,935	4,216,995	1,969,943 (46.71%)	1,308,192
i70_P1	7,355,781	6,359,647	4,434,881	2,003,227 (45.17%)	916,163
i70_P2	5,393,123	4,749,471	3,145,533	1,510,162 (48.01%)	1,064,134
i70_P3	8,474,438	7,503,466	5,112,916	2,394,936 (46.84%)	1,181,953
ER_P1	16,029,129	14,930,571	11,711,310	9,107,626 (77.77%)	1,773,615
ER_P2	12,685,198	11,597,086	8,132,570	5,814,301 (71.49%)	1,612,411
ER_P3	5,546,322	5,098,238	1,173,626	1,034,494 (88.15%)	244,939

* trim > 18 bp && < 32 bp (miRNA). #: number; QC: quality control; UMI: unique molecular identifiers; dedup.: deduplication; UC: ultracentrifugation; ES: ExoSpin; i35: qEV1 35 nm; i70: qEV1 70 nm; ER: ExoRNeasy; P1: pool#1; P2: pool#2; and P3: pool#3.

**Table 2:** Summary of the main properties of the different EV enrichment methods based on the downstream analyses performed in the present study.

	UC	ES	i35	i70	ER
Abundance of EVs surface markers	Variable across different detection techniques	Low	Medium	High	na
Abundance of EVs intravesicular markers	High	Low	High	High	na
Size distribution	Wide	Narrow	Narrow	Narrow	na
Size range (mode, mean; nm)	118, 168	113, 145	94, 129	80, 110	na
Protein cargo IDs	Low	High	Medium	High	na
miRNA cargo IDs	Medium	High	Low	Low	High
Carryover of serum proteins	High (mostly albumin)	High (mostly apolipoproteins)	Medium	Low	na
Costs/1 isolation	€	€€€	€€	€€	€€€
Time/1 isolation	5–6 h	2.5–3 h	1.5–2 h	1.5–2 h	1 h
Scalable	+++ *	++	-	-	+
Operator sensitive	+++	+	-	-	-

* depending on the available rotor. UC: differential ultracentrifugation; ES: ExoSpin; i35: qEV1 35nm; i70: qEV1 70 nm; ER: ExoRNeasy; na: not applicable; IDs: identifications.

## Data Availability

We have submitted all relevant data from our experiments to the EV-TRACK knowledgebase (EV-TRACK ID: EV250091) [22]. NGS raw data have been deposited in the European Nucleotide Archive (ENA) under the study accession number PRJEB94404. The mass spectrometry proteomics data have been deposited in the ProteomeXchange Consortium via the PRIDE [58] partner repository with the dataset identifier PXD068454.

## Supplementary Materials

The following supporting information can be downloaded at: https://www.mdpi.com/article/10.3390/ijms27020892/s1.

## Abbreviations

The following abbreviations are used in this manuscript:

EV	Extracellular Vesicles
UC	Ultracentrifugation
ES	ExoSpin
ER	ExoRNeasy
MVB	Multivesicular Body
ISEV	International Society for Extracellular Vesicles
i35	Izon qEV1–35nm
i70	Izon qEV1–75nm
TEM	Transmission Electron Microscopy
NTA	Nanoparticle Tracking Analysis
WB	Western Blot
NGS	Next-Generation Sequencing
FLOT-1	Flotillin-1
APO-AI	Apolipoprotein A-I
CANX	Calnexin
PCA	Principal Component Analysis
HCA	Hierarchical Clustering Analysis
GO	Gene Ontology
CC	Cellular Component
PDCD6	Programmed Cell Death Protein 6
ESCRT	Endosomal Sorting Complexes Required for Transport
SEC	Size Exclusion Chromatography
DTT	Dithiothreitol
DIA	Data-Independent Acquisition

## References

[1] Couch Y., Buzas E.I., Di Vizio D., Gho Y.S., Harrison P., Hill A.F., Lötvall J., Raposo G., Stahl P.D., Théry C. (2021). A brief history of nearly EV-erything—The rise and rise of extracellular vesicles. J. Extracell. Vesicles.

[2] Yates A.G., Pink R.C., Erdbrugger U., Siljander P.R., Dellar E.R., Pantazi P., Akbar N., Cooke W.R., Chir M.V.B.M., Dias-Neto E. (2022). In sickness and in health: The functional role of extracellular vesicles in physiology and pathology in vivo: Part I: Health and Normal Physiology: Part I: Health and Normal Physiology. J. Extracell. Vesicles.

[3] Cipriano M.J., Hajduk S.L. (2018). Drivers of persistent infection: Pathogen-induced extracellular vesicles. Essays Biochem..

[4] Munhoz da Rocha I.F., Amatuzzi R.F., Lucena A.C.R., Faoro H., Alves L.R. (2020). Cross-Kingdom Extracellular Vesicles EV-RNA Communication as a Mechanism for Host-Pathogen Interaction. Front. Cell. Infect. Microbiol..

[5] Rodrigues A., Weber J.I., Duraes-Oliveira J., Moreno C., Ferla M., Aires Pereira M., Valerio-Bolas A., Freitas B.E., Nunes T., Antunes W.T. (2025). Extracellular Vesicles Derived from Trypanosomatids: The Key to Decoding Host-Parasite Communication. Int. J. Mol. Sci..

[6] Tiberti N., Castilletti C., Gobbi F.G. (2025). Extracellular vesicles in arbovirus infections: From basic biology to potential clinical applications. Front. Cell. Infect. Microbiol..

[7] Buzas E.I. (2023). The roles of extracellular vesicles in the immune system. Nat. Rev. Immunol..

[8] Welsh J.A., Goberdhan D.C.I., O’Driscoll L., Buzas E.I., Blenkiron C., Bussolati B., Cai H., Di Vizio D., Driedonks T.A.P., Erdbrugger U. (2024). Minimal information for studies of extracellular vesicles (MISEV2023): From basic to advanced approaches. J. Extracell. Vesicles.

[9] Yu L., Shi H., Gao T., Xu W., Qian H., Jiang J., Yang X., Zhang X. (2025). Exomeres and supermeres: Current advances and perspectives. Bioact. Mater..

[10] Ozturk E.A., Caner A. (2022). Liquid Biopsy for Promising Non-invasive Diagnostic Biomarkers in Parasitic Infections. Acta Parasitol..

[11] Perry J.N., Jasim A., Hojat A., Yong W.H. (2019). Procurement, Storage, and Use of Blood in Biobanks. Methods Mol. Biol..

[12] Coppola L., Cianflone A., Grimaldi A.M., Incoronato M., Bevilacqua P., Messina F., Baselice S., Soricelli A., Mirabelli P., Salvatore M. (2019). Biobanking in health care: Evolution and future directions. J. Transl. Med..

[13] Litton J.E. (2018). Launch of an Infrastructure for Health Research: BBMRI-ERIC. Biopreservation Biobanking.

[14] Royo F., Thery C., Falcon-Perez J.M., Nieuwland R., Witwer K.W. (2020). Methods for Separation and Characterization of Extracellular Vesicles: Results of a Worldwide Survey Performed by the ISEV Rigor and Standardization Subcommittee. Cells.

[15] Lopez-Guerrero J.A., Vales-Gomez M., Borras F.E., Falcon-Perez J.M., Vicent M.J., Yanez-Mo M. (2023). Standardising the preanalytical reporting of biospecimens to improve reproducibility in extracellular vesicle research—A GEIVEX study. J. Extracell. Biol..

[16] Nieuwland R., Lucien F., Gustafson D., Lenassi M., Martinod K., Hisada Y. (2025). Monitoring and reporting the composition of plasma and serum to improve biobanks and comparability of extracellular vesicle research: Communication from the ISTH SSC Subcommittee on Vascular Biology. J. Thromb. Haemost. JTH.

[17] Lucien F., Gustafson D., Lenassi M., Li B., Teske J.J., Boilard E., von Hohenberg K.C., Falcon-Perez J.M., Gualerzi A., Reale A. (2023). MIBlood-EV: Minimal information to enhance the quality and reproducibility of blood extracellular vesicle research. J. Extracell. Vesicles.

[18] Baek R., Sondergaard E.K., Varming K., Jorgensen M.M. (2016). The impact of various preanalytical treatments on the phenotype of small extracellular vesicles in blood analyzed by protein microarray. J. Immunol. Methods.

[19] Malys M.S., Koller M.C., Papp K., Aigner C., Dioso D., Mucher P., Schachner H., Bonelli M., Haslacher H., Rees A.J. (2023). Small extracellular vesicles are released ex vivo from platelets into serum and from residual blood cells into stored plasma. J. Extracell. Biol..

[20] Taha H.B. (2023). Plasma versus serum for extracellular vesicle (EV) isolation: A duel for reproducibility and accuracy for CNS-originating EVs biomarker analysis. J. Neurosci. Res..

[21] Roux Q., Van Deun J., Dedeyne S., Hendrix A. (2020). The EV-TRACK summary add-on: Integration of experimental information in databases to ensure comprehensive interpretation of biological knowledge on extracellular vesicles. J. Extracell. Vesicles.

[22] Consortium E.-T., Van Deun J., Mestdagh P., Agostinis P., Akay O., Anand S., Anckaert J., Martinez Z.A., Baetens T., Beghein E. (2017). EV-TRACK: Transparent reporting and centralizing knowledge in extracellular vesicle research. Nat. Methods.

[23] Schorey J.S., Harding C.V. (2016). Extracellular vesicles and infectious diseases: New complexity to an old story. J. Clin. Investig..

[24] Brennan K., Martin K., FitzGerald S.P., O’Sullivan J., Wu Y., Blanco A., Richardson C., Mc Gee M.M. (2020). A comparison of methods for the isolation and separation of extracellular vesicles from protein and lipid particles in human serum. Sci. Rep..

[25] Bharti R., Kumar M., Devi V., Rao A., Aggarwal A., Gupta T. (2025). Efficient isolation and characterization of Serum-Derived Exosomes: Evaluating ultracentrifugation and Total Exosome Isolation Reagent based precipitation. Ultrastruct. Pathol..

[26] Clos-Sansalvador M., Monguio-Tortajada M., Roura S., Franquesa M., Borras F.E. (2022). Commonly used methods for extracellular vesicles’ enrichment: Implications in downstream analyses and use. Eur. J. Cell Biol..

[27] Veerman R.E., Teeuwen L., Czarnewski P., Gucluler Akpinar G., Sandberg A., Cao X., Pernemalm M., Orre L.M., Gabrielsson S., Eldh M. (2021). Molecular evaluation of five different isolation methods for extracellular vesicles reveals different clinical applicability and subcellular origin. J. Extracell. Vesicles.

[28] Takov K., Yellon D.M., Davidson S.M. (2019). Comparison of small extracellular vesicles isolated from plasma by ultracentrifugation or size-exclusion chromatography: Yield, purity and functional potential. J. Extracell. Vesicles.

[29] Bodin S., Elhabashy H., Macdonald E., Winter D., Gauthier-Rouviere C. (2025). Flotillins in membrane trafficking and physiopathology. Biol. Cell.

[30] Kim D.K., Lee J., Kim S.R., Choi D.S., Yoon Y.J., Kim J.H., Go G., Nhung D., Hong K., Jang S.C. (2015). EVpedia: A community web portal for extracellular vesicles research. Bioinformatics.

[31] Maki M., Takahara T., Shibata H. (2016). Multifaceted Roles of ALG-2 in Ca^2+^-Regulated Membrane Trafficking. Int. J. Mol. Sci..

[32] Lee K.M., Seo E.C., Lee J.H., Kim H.J., Hwangbo C. (2023). The Multifunctional Protein Syntenin-1: Regulator of Exosome Biogenesis, Cellular Function, and Tumor Progression. Int. J. Mol. Sci..

[33] Hagiwara K., Katsuda T., Gailhouste L., Kosaka N., Ochiya T. (2015). Commitment of Annexin A2 in recruitment of microRNAs into extracellular vesicles. FEBS Lett..

[34] Mulcahy L.A., Pink R.C., Carter D.R. (2014). Routes and mechanisms of extracellular vesicle uptake. J. Extracell. Vesicles.

[35] Colombo M., Raposo G., Thery C. (2014). Biogenesis, secretion, and intercellular interactions of exosomes and other extracellular vesicles. Annu. Rev. Cell Dev. Biol..

[36] Cheng L., Sun X., Scicluna B.J., Coleman B.M., Hill A.F. (2014). Characterization and deep sequencing analysis of exosomal and non-exosomal miRNA in human urine. Kidney Int..

[37] Chevillet J.R., Kang Q., Ruf I.K., Briggs H.A., Vojtech L.N., Hughes S.M., Cheng H.H., Arroyo J.D., Meredith E.K., Gallichotte E.N. (2014). Quantitative and stoichiometric analysis of the microRNA content of exosomes. Proc. Natl. Acad. Sci. USA.

[38] Jenike A.E., Halushka M.K. (2021). miR-21: A non-specific biomarker of all maladies. Biomark. Res..

[39] Visconte C., Fenoglio C., Serpente M., Muti P., Sacconi A., Rigoni M., Arighi A., Borracci V., Arcaro M., Arosio B. (2023). Altered Extracellular Vesicle miRNA Profile in Prodromal Alzheimer’s Disease. Int. J. Mol. Sci..

[40] Aparicio-Puerta E., Hirsch P., Schmartz G.P., Kern F., Fehlmann T., Keller A. (2023). miEAA 2023: Updates, new functional microRNA sets and improved enrichment visualizations. Nucleic Acids Res..

[41] Tzaridis T., Bachurski D., Liu S., Surmann K., Babatz F., Gesell Salazar M., Volker U., Hallek M., Herrlinger U., Vorberg I. (2021). Extracellular Vesicle Separation Techniques Impact Results from Human Blood Samples: Considerations for Diagnostic Applications. Int. J. Mol. Sci..

[42] Linares R., Tan S., Gounou C., Arraud N., Brisson A.R. (2015). High-speed centrifugation induces aggregation of extracellular vesicles. J. Extracell. Vesicles.

[43] Van Deun J., Mestdagh P., Sormunen R., Cocquyt V., Vermaelen K., Vandesompele J., Bracke M., De Wever O., Hendrix A. (2014). The impact of disparate isolation methods for extracellular vesicles on downstream RNA profiling. J. Extracell. Vesicles.

[44] Karimi N., Dalirfardouei R., Dias T., Lotvall J., Lasser C. (2022). Tetraspanins distinguish separate extracellular vesicle subpopulations in human serum and plasma—Contributions of platelet extracellular vesicles in plasma samples. J. Extracell. Vesicles.

[45] Xu D., Di K., Fan B., Wu J., Gu X., Sun Y., Khan A., Li P., Li Z. (2022). MicroRNAs in extracellular vesicles: Sorting mechanisms, diagnostic value, isolation, and detection technology. Front. Bioeng. Biotechnol..

[46] Lamorte D., De Luca L., Tartarone A., Trino S., Giulivo I., De Stradis A., Maietti M., Caivano A., Laurenzana I. (2025). Serum extracellular vesicle microRNAs as potential biomarkers to predict pembrolizumab response and prognosis in metastatic non-small cell lung cancer patients. Front. Immunol..

[47] Zhou H., Gong J., Zhang Z., Wang B., Qiu Y., Xu H., Sun X., Li Z. (2025). Proteomic Profiling of Serum-Derived Extracellular Vesicles in Diffuse Idiopathic Skeletal Hyperostosis Patients. J. Proteome Res..

[48] Dhondt B., Pinheiro C., Geeurickx E., Tulkens J., Vergauwen G., Van Der Pol E., Nieuwland R., Decock A., Miinalainen I., Rappu P. (2023). Benchmarking blood collection tubes and processing intervals for extracellular vesicle performance metrics. J. Extracell. Vesicles.

[49] Burrello J., Monticone S., Burrello A., Bolis S., Cristalli C.P., Comai G., Corradetti V., Grange C., Orlando G., Bonafe M. (2023). Identification of a serum and urine extracellular vesicle signature predicting renal outcome after kidney transplant. Nephrol. Dial. Transplant. Off. Publ. Eur. Dial. Transpl. Assoc.—Eur. Ren. Assoc..

[50] Tran V., de Oliveira G.P., Chidester S., Lu S., Pleet M.L., Ivanov A.R., Tigges J., Yang M., Jacobson S., Goncalves M.C.B. (2024). Choice of blood collection methods influences extracellular vesicles counts and miRNA profiling. J. Extracell. Biol..

[51] Yang C., Han J., Liu H., He Y., Zhang Z., Liu X., Waqas F., Zhang L., Duan H., He J. (2024). Storage of plasma-derived exosomes: Evaluation of anticoagulant use and preserving temperatures. Platelets.

[52] Chang C.J., Huang Y.N., Lu Y.B., Zhang Y., Wu P.H., Huang J.S., Yang W., Chiang T.Y., Hsieh H.S., Chung W.H. (2024). Proteomic analysis of serum extracellular vesicles from biliary tract infection patients to identify novel biomarkers. Sci. Rep..

[53] Buschmann D., Kirchner B., Hermann S., Marte M., Wurmser C., Brandes F., Kotschote S., Bonin M., Steinlein O.K., Pfaffl M.W. (2018). Evaluation of serum extracellular vesicle isolation methods for profiling miRNAs by next-generation sequencing. J. Extracell. Vesicles.

[54] Quek C., Bellingham S.A., Jung C.H., Scicluna B.J., Shambrook M.C., Sharples R.A., Cheng L., Hill A.F. (2017). Defining the purity of exosomes required for diagnostic profiling of small RNA suitable for biomarker discovery. RNA Biol..

[55] Ekstrom K., Crescitelli R., Petursson H.I., Johansson J., Lasser C., Olofsson Bagge R. (2022). Characterization of surface markers on extracellular vesicles isolated from lymphatic exudate from patients with breast cancer. BMC Cancer.

[56] Huang D.W., Sherman B.T., Lempicki R.A. (2009). Systematic and integrative analysis of large gene lists using DAVID bioinformatics resources. Nat. Protoc..

[57] Sherman B.T., Hao M., Qiu J., Jiao X., Baseler M.W., Lane H.C., Imamichi T., Chang W. (2022). DAVID: A web server for functional enrichment analysis and functional annotation of gene lists (2021 update). Nucleic Acids Res..

[58] Perez-Riverol Y., Csordas A., Bai J., Bernal-Llinares M., Hewapathirana S., Kundu D.J., Inuganti A., Griss J., Mayer G., Eisenacher M. (2019). The PRIDE database and related tools and resources in 2019: Improving support for quantification data. Nucleic Acids Res..

