# Supplementary material for: Molecular Evaluation of Different Enrichment Methods for Extracellular Vesicles from Healthy Subjects’ Biobanked Serum
Source: Int J Mol Sci. 2026 Jan 15;27(2):892. doi: 10.3390/ijms27020892 (PMC12842214; doi:10.3390/ijms27020892)

SUPPLEMENTARY FIGURES

Molecular evaluation of different enrichment methods of extracellular vesicles from biobanked serum from healthy subjects

Michela Deiana[1], Elisabetta Vezzelli[1], Cristina Mazzi[2], Denise Lavezzari[1], Marcello Manfredi[3,4], Francesca Moretta[5], Chiara Piubelli[1], Federico Giovanni Gobbi[1,6], Natalia Tiberti[1*]

1 Department of Infectious, Tropical Diseases and Microbiology, IRCCS Sacro Cuore Don Calabria Hospital, Negrar di Valpolicella (Verona), Italy

2 Clinical Research Unit, IRCCS Sacro Cuore Don Calabria Hospital, Negrar di Valpolicella (Verona), Italy

3 Department of Translational Medicine, University of Piemonte Orientale, Novara, Italy

4 Institute for Molecular and Translational Cardiology (IMTC), IRCCS Policlinico San Donato, S. Donato Milanese (Milano), Italy

5 Clinical Analysis Laboratory and Transfusional Medicine, Clinical Pharmacology, IRCCS Sacro Cuore Don Calabria Hospital, Negrar di Valpolicella (Verona), Italy

6 Department of Clinical and Experimental Sciences, University of Brescia, Brescia, Italy

Supplementary Figure S1. Entire images of the western blot experiments performed to assess the presence of APO-AI in the three pools of serum samples (P1, P2, P3) used to enriched EVs.

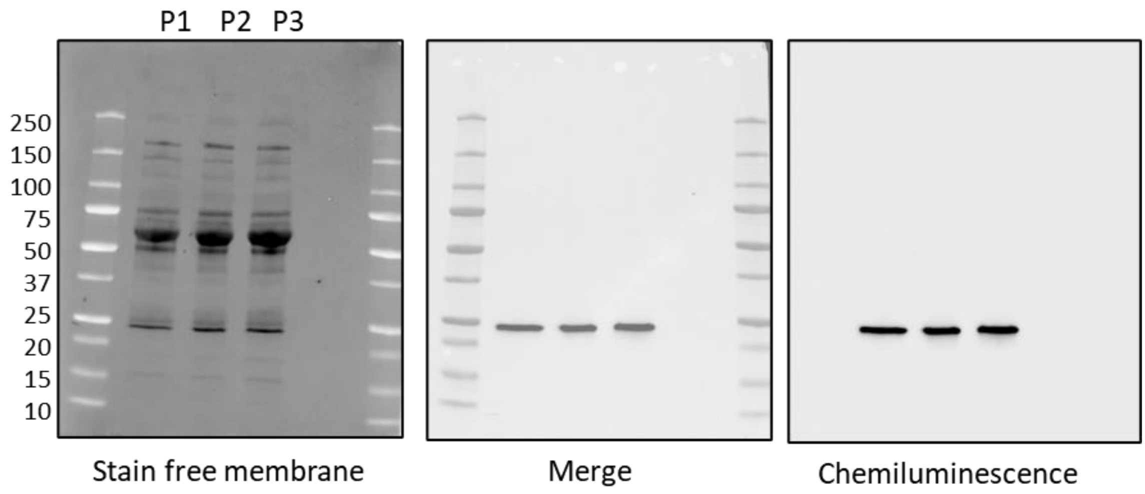

APO-AI 0.2µg/mL, in 5% non-fat milk
Goat anti-rabbit immunoglobulins/HRP 1/2000
Predicted size 27-31 kDa

Supplementary Figure S2 A-E. Entire images of the western blot experiments presented in the main manuscript. For each protein target, the stain free membrane used to obtain relative band quantification, the merged image of colorimetric and chemiluminescent signals and the chemiluminescent signal are reported.

A) APOLIPOPROTEIN-AI

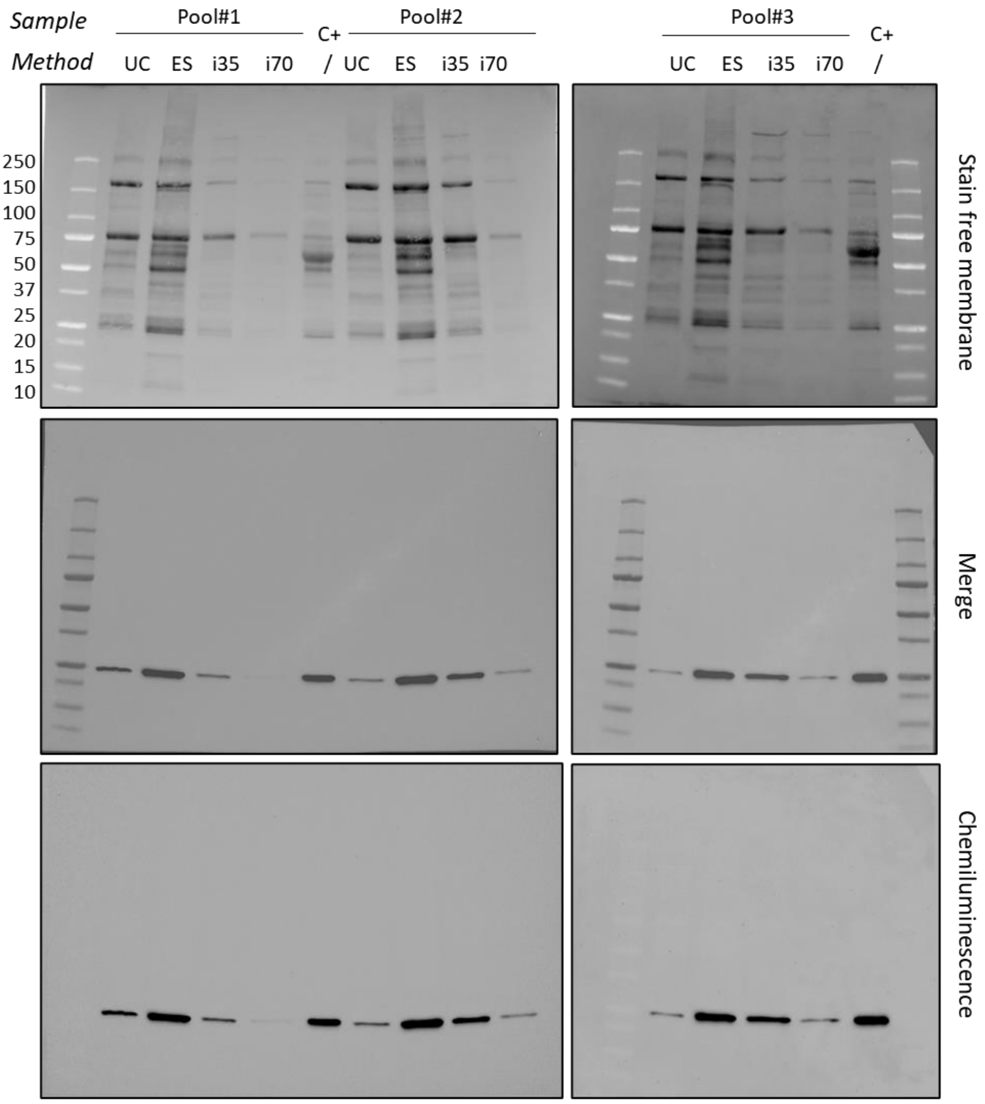

APO-AI 0.2µg/mL, in 5% non-fat milk
Goat anti-rabbit immunoglobulins/HRP 1/2000
Predicted size 27-31 kDa

B) FLOTILLIN-1

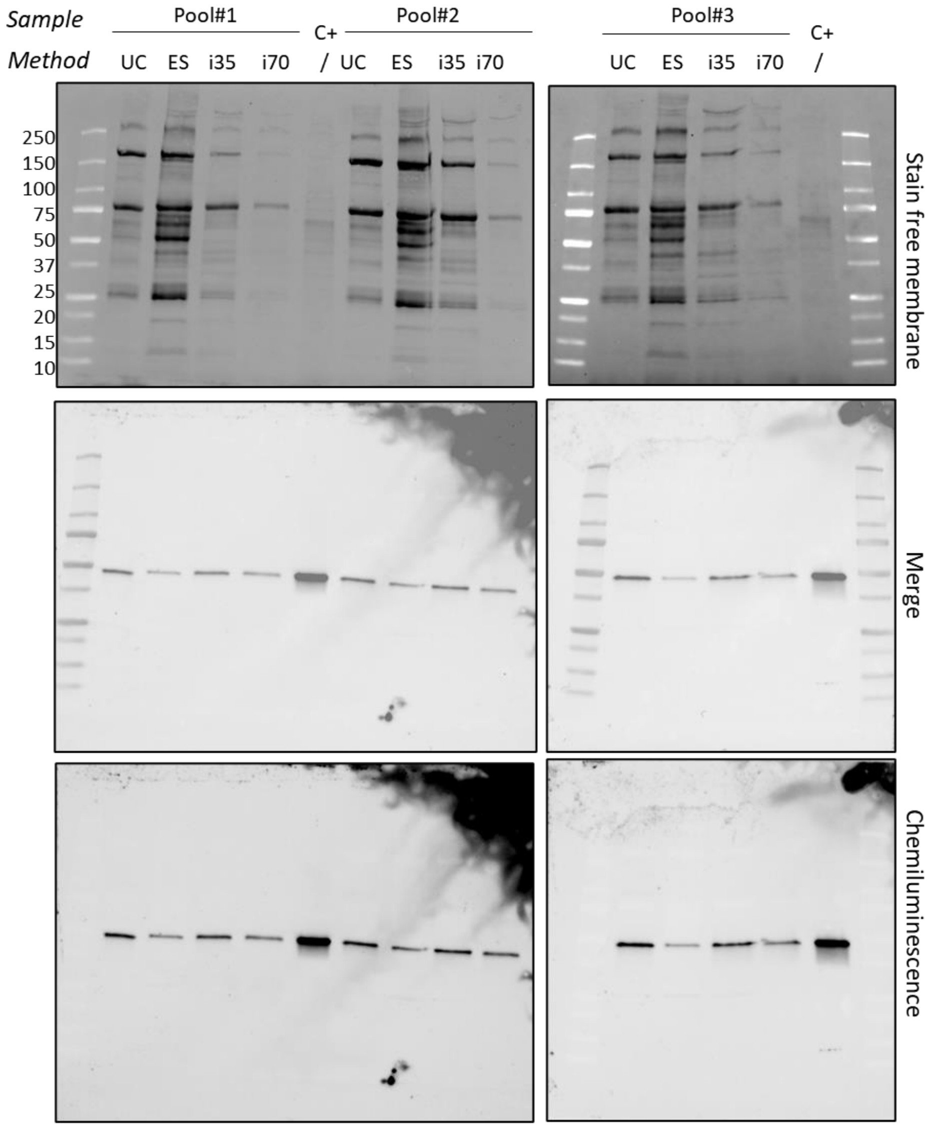

FLOT-1 1µg/mL, in 5% non-fat milk
Goat anti-rabbit immunoglobulins/HRP 1/2000
Predicted size 47 kDa

C) CD63

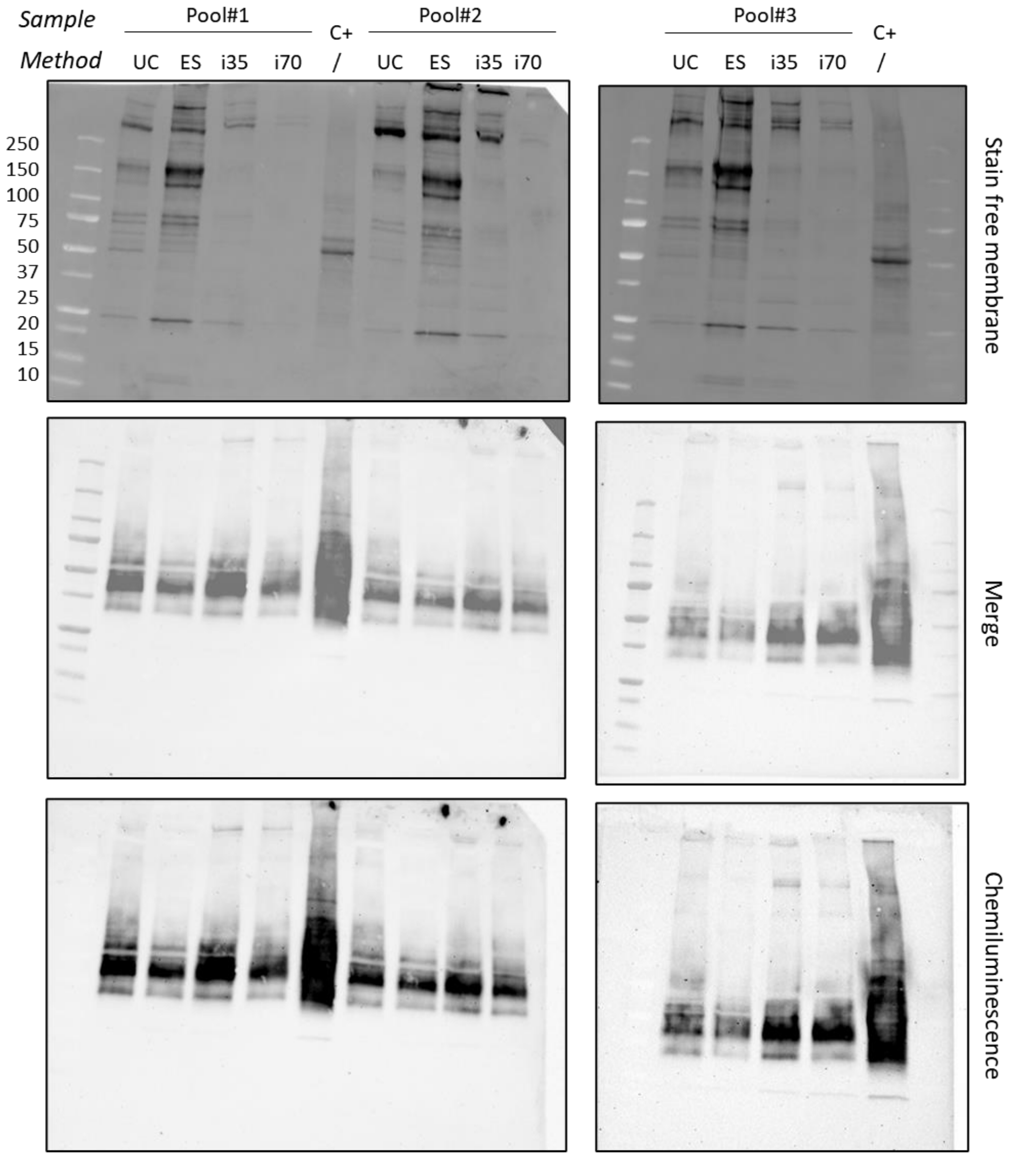

CD63 1µg/mL, in 5% non-fat milk
Goat anti-mouse immunoglobulins/HRP 1/1500
Non-reducing conditions
Predicted size 30-60 kDa

D) CD81

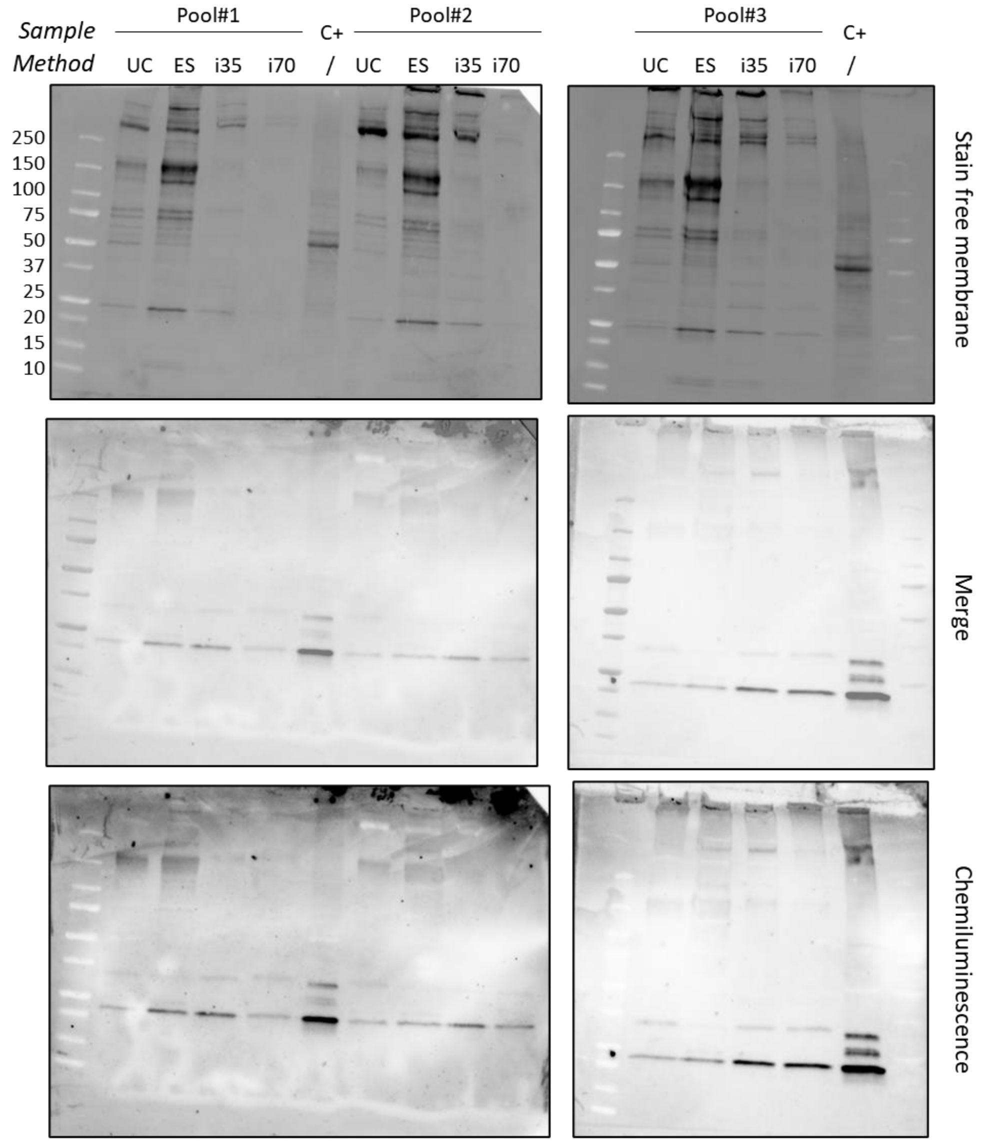

CD81 2µg/mL, in 5% non-fat milk
Goat anti-mouse immunoglobulins/HRP 1/1500
Non-reducing conditions
Predicted size 25 kDa

E) CALNEXIN

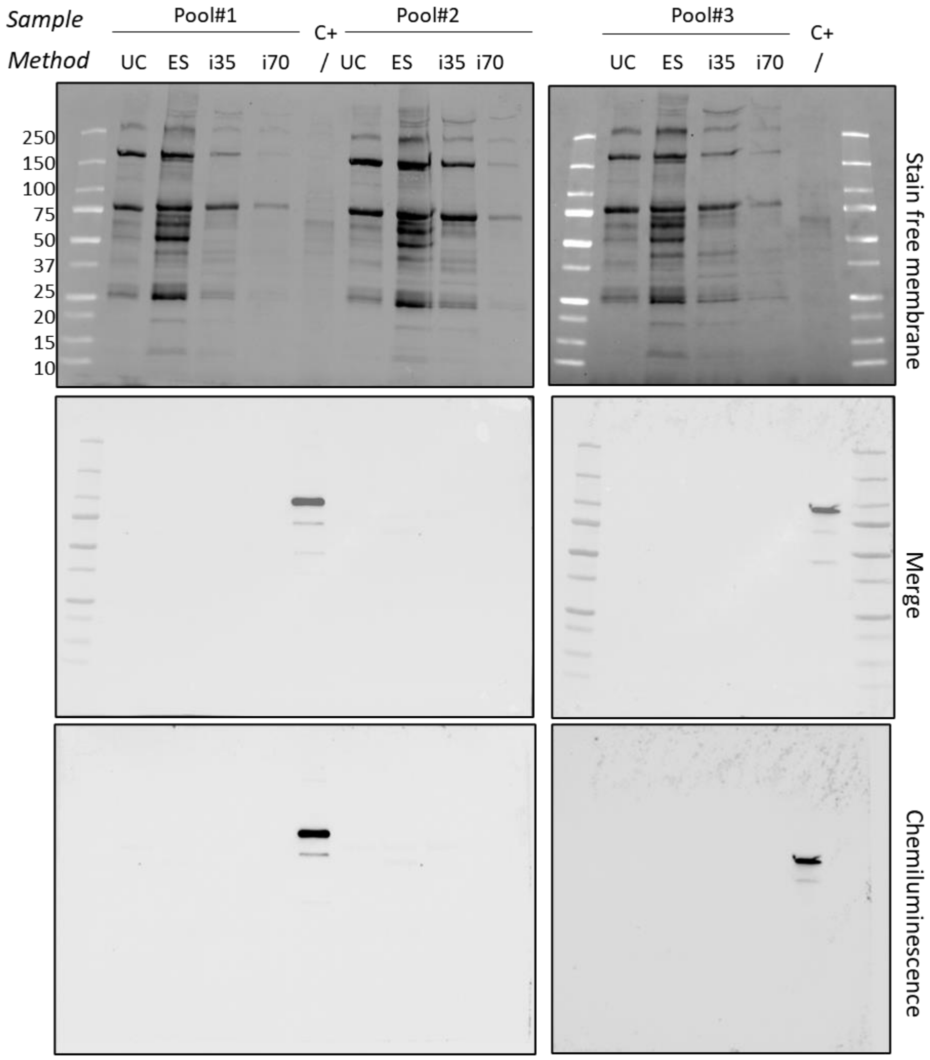

CANX 1µg/mL, in 5% non-fat milk
Goat anti-mouse immunoglobulins/HRP 1/1500
Predicted size 68 kDa

Supplementary Figure S3. Proteomics results. A) Venn diagram comparing the proteins identified in EV samples prepared with the different enrichment methods. (diagrams obtained with https://bioinfogp.cnb.csic.es/tools/venny/). B, C) Proportion of immuno-depleted proteins identified in EV prepared with the different methods and in the 3 biological samples. Proportions were calculated for each individual sample dividing the normalised abundances to the total protein abundances.

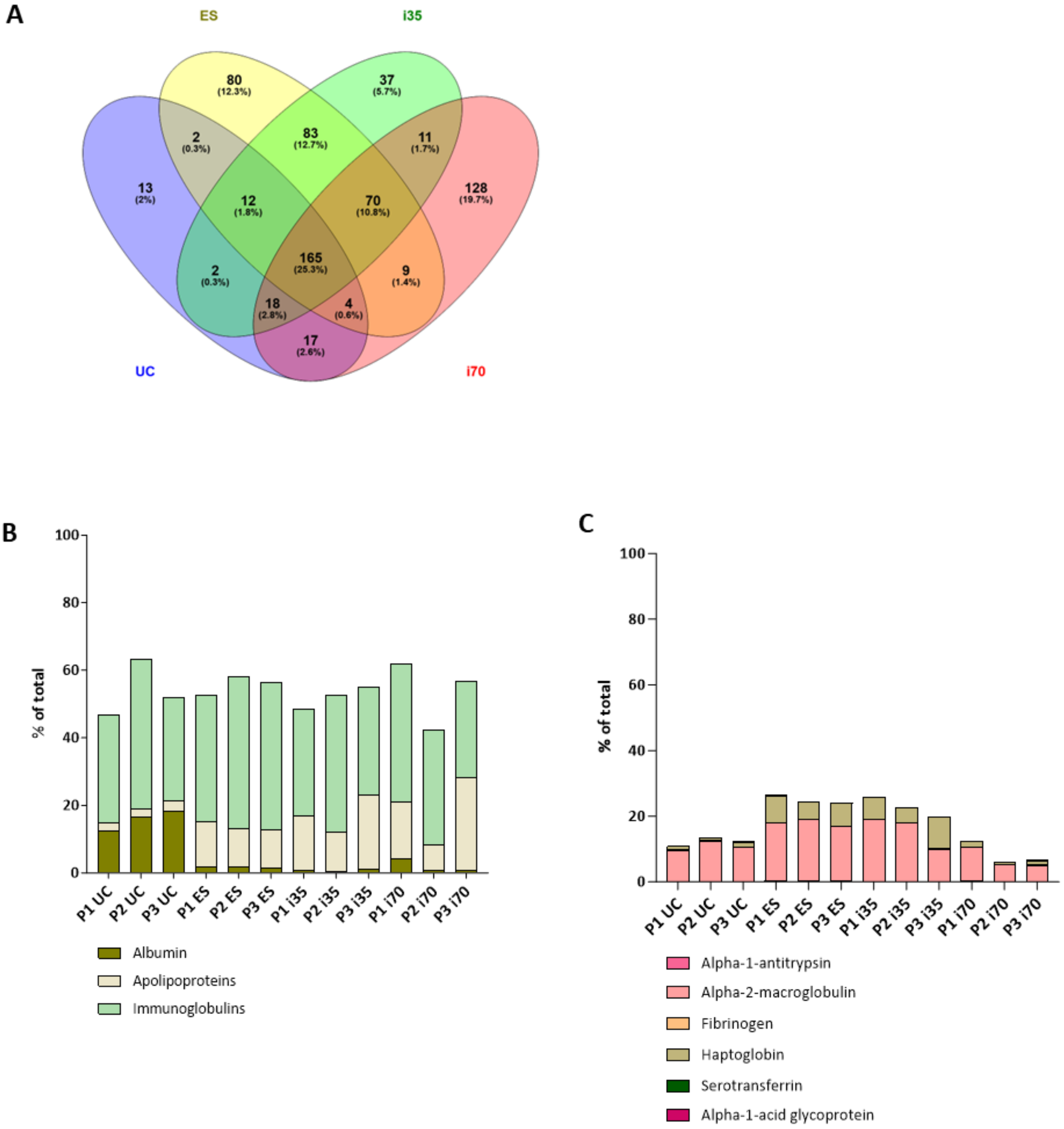

Supplementary Figure S4. NGS results A) Representative TapeStation electropherograms of total RNA isolated from EVs obtained using the five isolation methods. The x-axis represents RNA fragment size (nt), and the y-axis shows fluorescence intensity (FU). The peak at 25 nt corresponds to the internal marker. B) Quantification of total RNA isolated (in ng) for each method. In the table are reported for each sample, the RNA concentration, minimum and maximum fragment size and the total RNA yield (ng) obtained from EVs derived from 250 µL of serum. C) Venn diagram comparing the miRNA sequences detected in EV samples prepared with UC, ES and ER. Izons-preparations were excluded due to the low number of identified miRNAs (diagrams obtained with https://bioinfogp.cnb.csic.es/tools/venny/).

A

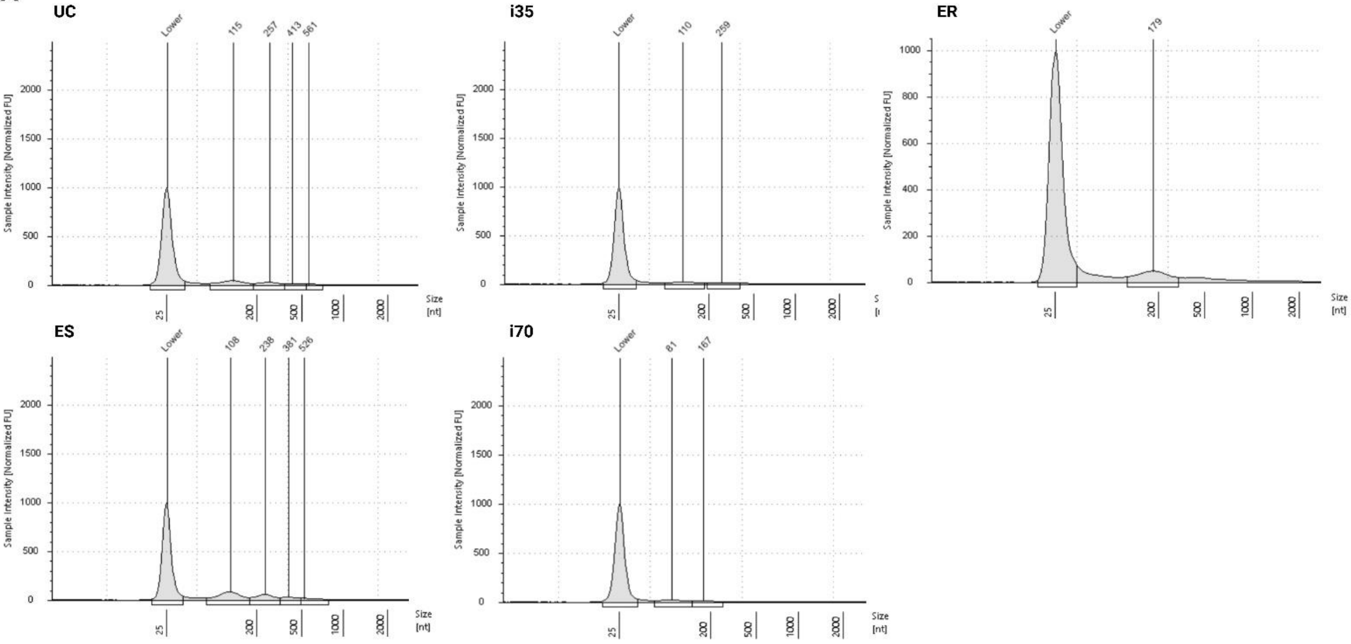

B

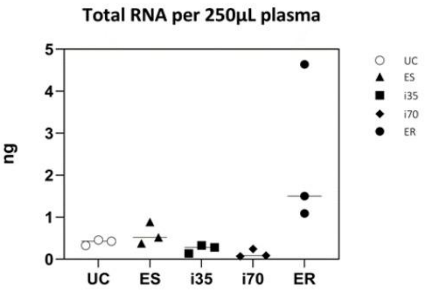

Sample	pg/µl	bp_min	bp_max	ng total
UC_P1	104.0	25	237	0.55
UC_P2	177.0	25	249	0.88
UC_P3	75.4	25	228	0.37
ES_P1	65.0	25	224	0.32
ES_P2	93.1	25	245	0.46
ES_P3	85.5	25	236	0.47
i35_P1	27.2	25	163	0.13
i35_P2	65.3	25	246	0.32
i35_P3	56.4	25	220	0.28
i70_P1	12.2	25	80	0.08
i70_P2	13.9	25	65	0.06
i70_P3	49.1	25	146	0.24
ER_P1	179.2	25	751	0.89
ER_P2	259.2	25	1085	1.29
ER_P3	824.4	25	1084	4.12

C

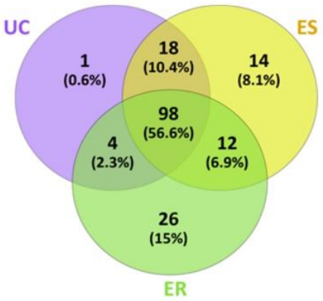

Supplement: Supplementary file 1 [file ijms-27-00892-s001.zip › Deiana_et_al_Supplementary_Figures.pdf]
