# Supplementary material for: Molecular Evaluation of Different Enrichment Methods for Extracellular Vesicles from Healthy Subjects’ Biobanked Serum
Source: Int J Mol Sci. 2026 Jan 15;27(2):892. doi: 10.3390/ijms27020892 (PMC12842214; doi:10.3390/ijms27020892)
Supplement: Supplementary file 1 [file ijms-27-00892-s001.zip › Supplementary_File_S2.pdf]

INTERNATIONAL SOCIETY *for*
EXTRACELLULAR VESICLES

MIBlood-EV

Standardized Reporting Tool for Blood EV Research (Human)

STUDY INFORMATION

**1.0 Manuscript title**	Molecular evaluation of different enrichment methods of extracellular vesicles from bio-banked serum from healthy subjects
**1.1 Corresponding author (Name and Email)**	Natalia Tiberti, natalia.tiberti@sacrocuore.it
**1.2 Institution name**	IRCCS Sacro Cuore Don Calabria Hospital
**1.3 Time period of experiment (e.g. 2022-2024)**	2024-2025
**1.4 Number of samples**	3 pools of 5 samples
**1.5 Cargo of interest**	☑ Vesicles ☑ Protein ☑ RNA ☐ DNA ☐ Other:
**1.6 Biospecimen type**	☐ Plasma ☑ Serum
**1.7 Biospecimen state**	Frozen
**1.8 Source of frozen specimens**	Obtained from biobank
**1.9 Years of collection (range)**	2020-2022

BLOOD COLLECTION AND PROCESSING

**2.0 Patient fasting status**	Uncertain
**2.1 Fasting length (e.g. hours/days)**	unknown
**2.2 Anatomical access site**	unknown
**2.3 Needle diameter (e.g. gauge)**	21G
**2.4 Blood volume collected (mL)**	3x6 ml
**2.5 Plasma anticoagulant**	☐ EDTA ☐ Citrate ☐ Heparin ☐ Other:
**2.6 Serum tube type**	Clot activator + Gel separator (SST)
**2.7 Serum clotting time (minutes)**	unknown
**2.8 Time between collection and first centrifugation (range in hours)**	2-8
**2.9 Transport temperature**	Room temp (~22°C)
**2.10 Transport condition of tubes**	Vertical
**2.11 Centrifuge brand and model**	ALC refrigerated centrifuge
**2.12 Bucket rotor type**	Swing
**2.13 Number of centrifugation cycles**	One
**2.14a 1st Centrifugation speed (RCF in x g)**	1650
**2.14b 1st Centrifugation time (minutes)**	8
**2.15 1st Rotor brake**	Off
**2.16 1st Centrifugation temperature**	Room temp (~22°C)
**2.17a 2nd Centrifugation speed (RCF in x g)**	
**2.17b 2nd Centrifugation time (minutes)**	
**2.18 2nd Rotor brake**	
**2.19 2nd Centrifugation temperature**	
**2.20 Additional processing steps (e.g. filtration)**	None
**2.21 Storage tubes (brand, type, source, catalog number)**	1.5ml micro-tube, PP, trasparent, Sarstedt (Ref: 72.690.001)
**2.22 Storage temperature**	-80°C
**2.23 Length of storage (range in years)**	3-5

PLASMA/SERUM QUALITY CONTROL

**3.0 Number of freeze-thaw cycles (range)**	1
**3.1 Thawing temperature**	Room Temp (~22°C)
**3.2 Thawing duration (minutes)**	<5

Hemolysis

**3.3 Presence of hemolysis**	No
**3.4 Frequency of hemolyzed samples (e.g. <25%, 25-50%)**	0
**3.5 Method used**	Visual inspection
**3.6 RBC count (Median, 95% CI, N)**	See attached table
**3.7 RBC counter brand and type**	Sysmex XR-Series Automated Hematology Analyzer XR-1000/XR-2000, LoD: 0.002 x 10^6/µL
**3.8 Spectrophotometry hemoglobin concentration (mean g/L)**	
**3.9 Spectrophotometer brand, model and wavelength measured (e.g. 414 nm)**	
**3.10 Hemolized samples were discarded**	

Platelets

3.11 **Presence of platelets**	Yes	3.12 **Method used (e.g. Flow Cytometry)**	Hematology Analyzer
3.13 **Marker(s) used (e.g. CD61, CD41)**			
3.14 **Concentration (median, 95% CI, N)**	See file attached		
3.15 **Platelet counter instrument brand, type and limit of detection (cells/L)**	Sysmex XR-Series Automated Hematology Analyzer XR-1000/XR-2000. LoD: 2 x 10^3/uL		
3.16 **Flow cytometer brand and type**			
3.17 **Flow cytometry size and fluorescence ranges of detection in nanometers and MESF, respectively**			

Lipoproteins

3.18 **Presence of lipoproteins**	Yes	3.19 **Method used (WB, ELISA, FC)**	Western-blot
3.20 **Spectrophotometry L-index**			
3.21 **Spectrophotometer brand, model and wavelength measured (e.g. 700 nm)**			
3.22 **WB Marker(s) used (e.g. Apo B)**	Apo AI		
3.23 **Western blot images provided in manuscript?**	Yes		
3.24 **Flow cytometry marker(s) used (e.g. ApoB)**			
3.25 **Flow cytometry concentration (median, 95% CI, N)**			
3.26 **Flow cytometer brand and type**			
3.27 **Flow cytometry size and fluorescence ranges of detection in nanometers and MESF, respectively**			
3.28 **Brand and catalog number for ELISA kit used for each Apolipoprotein test (e.g. ApoB)**			
3.29 **Concentration measured for each Apolipoprotein tested (mean µg/mL)**			
